# Exposure to Per- and Polyfluoroalkyl Substances (PFAS) Causes Dental Developmental Anomalies: Underrecognized Risk of Fluoride Bioaccumulation

**DOI:** 10.1021/acs.est.5c18166

**Published:** 2026-06-22

**Authors:** Motoki Okamoto, Shohei Yamashita, Nanako Kuriki, Susanne Brueckner, Ria Achong-Bowe, Melanie Mendonca, Juliana Sanches Trevizol, Natsumi Fujiwara, Shin Nakamura, Satoru Shindo, Takumi Memida, Manabu Mizuhira, Navi Gill Dhillon, Xiaozhe Han, Toshihisa Kawai, Marília Afonso Rabelo Buzalaf, Eric T. Everett, Maiko Suzuki

**Affiliations:** † Department of Oral Science and Translational Research, College of Dental Medicine, 2814 Nova Southeastern University, Fort Lauderdale, Florida 33314, United States; ‡ Department of Restorative Dentistry and Endodontology, Osaka University Graduate School of Dentistry, Suita, Osaka 565-0871, Japan; § Department of Oral Health Care Management, Graduate School of Biomedical Sciences, Tokushima University, Kuramoto, Tokushima 770-8504, Japan; ∥ Bruker Japan K. K. Nano Analytics Division, 3-9 Moriyacho, Yokohama, Kanagawa 221-0022, Japan; ⊥ Biology I Halmos College of Arts and Sciences, Behavioral Neuroscience I College of Psychology, 2814 Nova Southeastern University, Fort Lauderdale, Florida 33314, United States; # Department of Biological Sciences, Bauru School of Dentistry, University of Sao Paulo, Bauru, 17012-902, Brazil; ¶ Department of Biomedical Sciences, Adams School of Dentistry, 2331 The University of North Carolina at Chapel Hill, Chapel Hill, North Carolina 27599, United States

**Keywords:** PFAS, FTOH, PFOA, fluoride, bioaccumulation, odontogenesis, hypoplasia

## Abstract

Per- and polyfluoroalkyl substances (PFAS) are synthetic chemicals that persist in the environment and have potential health risks. Among them, 8:2 fluorotelomer alcohol (FTOH) undergoes microbial biotransformation in the environment into perfluorooctanoic acid (PFOA) and releases fluoride. While PFOA’s toxic effects have been well-studied, the impact of PFOA (and its precursor 8:2 FTOH) on dental health is largely unknown. Moreover, it is not established whether, and to what extent, defluorination and fluoride bioaccumulation occur during the *in vivo* 8:2 FTOH metabolism. This study is the first to comprehensively demonstrate the pathophysiology of PFAS-associated developmental dental anomalies—enamel and dentin hypoplasia—in the context of fluoride bioaccumulation after exposure to 8:2 FTOH in mice. Over 90 days, mice (male and female C57BL/6J) received daily oral doses of 8:2 FTOH. At the high dose, the levels of PFOA and 7:3 FTCA (the main 8:2 FTOH metabolites) in blood increased significantly, reaching PFOA concentrations comparable to those in occupationally exposed humans, underscoring the relevance of high-exposure scenarios. Fluoride levels significantly increased in the blood, urine, and bone, approaching levels linked to dental fluorosis in animal models. Dental defects included enamel and dentin hypoplasia, discoloration, reduced mineral density, and structural abnormalities, including damaged ameloblasts and immature mineral composition. Although some features resembled fluorosis, the defects were distinct. 8:2 FTOH and other PFAS capable of similar metabolic conversion may represent an understudied source of fluorine accumulation and a potential, previously unrecognized contributor to cryptogenic odontogenic abnormalities.

## Graphical abstract

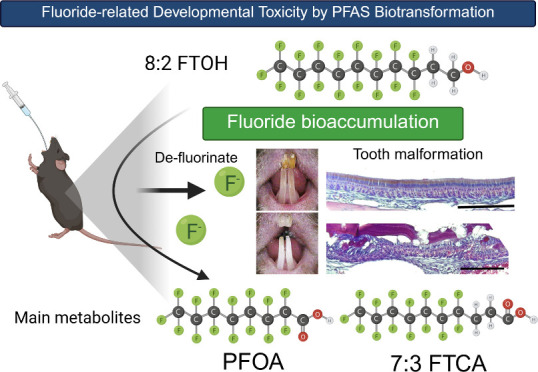

## 1 Introduction

Per- and polyfluoroalkyl substances (PFAS) are a large and diverse class of synthetic organofluoride compounds widely used in industrial and consumer products for their water-, stain-, and heat-resistant properties.^1–3^ PFAS are broadly categorized into per- and polyfluorinated compounds (Figure 1A). Among the most studied are perfluorooctanoic acid (PFOA) and perfluorooctanesulfonic acid (PFOS). Due to their environmental persistence and bioaccumulative properties, these compounds have been associated with adverse health outcomes, including hepatotoxicity,^4^ endocrine disruption,^5^ and neurodevelopmental toxicity.^6–9^ PFAS have been linked to developmental and oral health outcomes, yet only limited research has examined dental and craniofacial development specifically. Epidemiologic findings suggest craniofacial features may be PFAS-sensitive: in the Danish National Birth Cohort, prenatal PFAS exposure was associated with shorter palpebral fissure length at age 5.^10^ With respect to oral health, associations have been reported between PFAS and periodontitis (NHANES-based analyses showing positive relationships for PFOS and PFNA),^11^ and possible links to dental caries in children (e.g., perfluorodecanoic acid),^12^ underscoring potential oral end points of concern. Recent cohorts have also begun to evaluate developmental defects of enamel (DDE)/molar-incisor hypomineralization (MIH) in relation to PFAS or persistent organic pollutants, including sex-specific associations for PFOA/PFOS in French children^13^ and PFAS-specific effects on DDE in a Shanghai birth cohort.^14^ Experimental models further support craniofacial susceptibility, with PFOS/PFOA/PFNA causing morphometric and behavioral abnormalities in zebrafish embryos^15^ and PFOS inducing cleft palate in rodents at developmental exposures.^16,17^ We previously demonstrated that PFOA induces apoptosis and necroptosis in ameloblast-like cells (ALCs) in vitro, suggesting a direct cytotoxic effect on ameloblasts responsible for enamel formation.^18^

**Figure 1:**
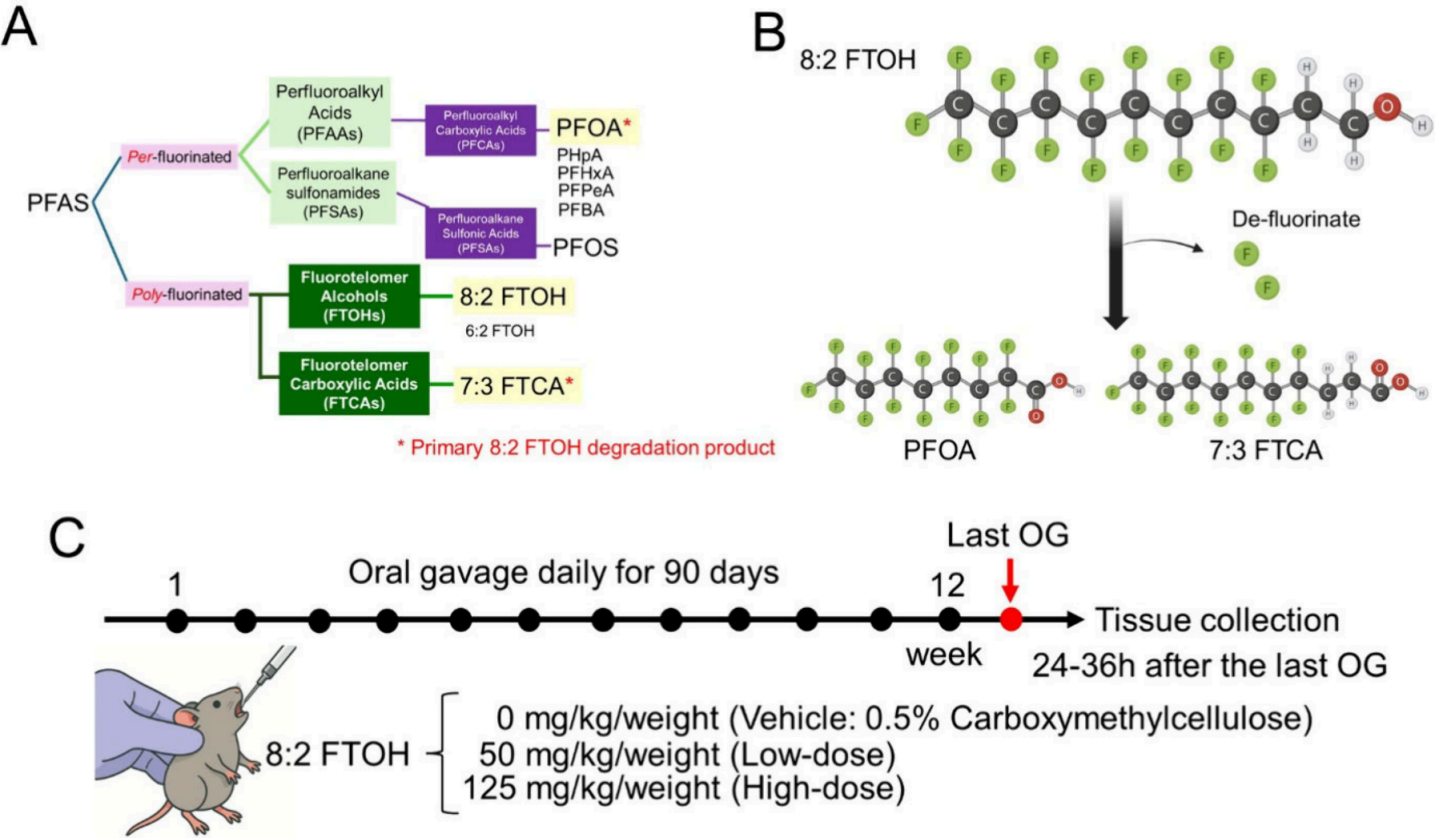
Overview of PFAS and Experimental Methods. (A) Classification of per- and polyfluoroalkyl substances (PFAS), a large group of synthetic organofluorine compounds. The panel shows representative PFAS compounds included in the present analysis, not exhaustive examples. This study focuses on 8:2 fluorotelomer alcohol (8:2 FTOH) and its primary *in vivo* metabolic product, 7:3 fluorotelomer carboxylic acid (7:3 FTCA) and perfluorooctanoic acid (PFOA). 8:2 FTOH is classified as a polyfluorinated PFAS, while PFOA is classified as a perfluorinated PFAS. (B) Simplified schematic hypothesis of *in vivo* defluorination: 8:2 FTOH undergoes multiple branching metabolic pathways with accompanying defluorination, generating intermediates such as 8:2 FTCA, 8:2 FTUCA, and 7:3 FTCA, with PFOA only one of several low-yield terminal acids produced *in vivo*. (C) Experimental schedule for 8:2 FTOH administration. Male (*N* = 6/group) and female (*N* = 7/group) mice were administered 8:2 FTOH daily by oral gavage (OG) for 90 days. Three groups were analyzed: Vehicle control (0 mg/kg body weight/day), low dose (50 mg/kg body weight/day), or high dose (125 mg/kg body weight/day) of 8:2 FTOH. Vehicle control: 0.5% carboxymethylcellulose. The doses of 8:2 FTOH were selected based on prior toxicological studies in animal models. At 24–36h after the last oral gavage (OG), tissues were collected for analysis. Our results confirmed that these doses resulted in plasma PFOA levels comparable to those observed in occupationally exposed individuals, supporting their relevance for modeling high-exposure human scenarios.

Although PFAS are known for their chemical stability, which is attributed to the strength of carbon–fluorine bonds,^19^ certain PFAS — like Fluorotelomer alcohols (FTOHs) — can be degraded in the environment^20–24^ and via metabolism. 8:2 FTOH is a volatile polyfluoroalkyl substance used in fluorotelomer-based polymers that are applied to textiles and consumer products.^25^ During manufacturing and product use, 8:2 FTOH can be released into indoor air, dust, wastewater, and soil, and environmental media, indicating a potential inhalation risk for the workers.^26^ Although human exposure to 8:2 FTOH occurs predominantly via inhalation of indoor/occupational air, oral intake of FTOHs can be another route of human exposure (e.g., dust ingestion, and food samples).^27–29^ However, no human toxicokinetic (TK) data exist for 8:2 FTOH. 8:2 FTOH is a precursor of PFOA and can undergo two distinct transformation contexts: (i) microbial biotransformation in the environment (e.g, sludge, soils) accompanied by defluorination, which informs potential sources of environmental PFOA and fluoride ion,^20–24^ and (ii) *in vivo* metabolism, where 8:2 FTOH is converted to PFOA and related acids (e.g., 7:3 FTCA) with low, incomplete yields in rodents^30–33^ and humans.^34,35^ 8:2 FTOH undergoes multiple oxidative and rearrangement steps to form intermediates such as 8:2 FTCA, 8:2 FTUCA, and 7:3 FTCA through metabolism by microsomal and cytosolic enzymes, including CYP2C19 and other microsomal CYPs,^36,37^ and ultimately yields only low levels of PFOA (<0.1%^36^ in rats and 2.5–5% in humans^34,35^ of the parent 8:2 FTOH).

Because FTOHs are volatile and undergo long-range transport, they are important environmental sources of PFOA,^38^ but — crucially for our work — 8:2 FTOH also can serve as an *in vivo* precursor that can elevate internal PFOA burdens. Although previous studies indicate that only low levels of 8:2 FTOH are metabolically converted to PFOA in humans^34,35^ — suggesting that 8:2 FTOH contributes minimally to overall PFOA exposure—the potential for fluoride ion generation from 8:2 FTOH in vivo remains largely uncharacterized. Importantly, aside from environmental microbial biodegradation, it is not yet established whether, and to what extent, defluorination occurs during the *in vivo* metabolism of 8:2 FTOH.

Fluoride at optimal levels is essential for dental health and promotes enamel remineralization and caries prevention.^39^ However, excessive fluoride exposure is a recognized public health concern associated with dental and skeletal fluorosis.^40^ During tooth development, fluoride overexposure can lead to bioaccumulation of fluoride in mineralized tissues, resulting in dental fluorosis, characterized by enamel and dentin hypomineralization, structural defects, and altered mineral composition.^41,42^ The developing dentition is particularly vulnerable to environmental toxicants because enamel and dentin formation rely on tightly regulated cellular and molecular processes. Although several studies have reported FTOHs-induced tooth malformations in rodents following exposure to 6:2 FTOH, 8:2 FTOH, 10:2 FTOH, and their mixtures,^43–46^ these investigations did not demonstrate the specific tooth phenotypic, microstructural, and histological characteristics of dental defects. The underlying mechanisms remain even less well understood, highlighting a critical knowledge gap.

To address this gap, we hypothesized that chronic 8:2 FTOH exposure in rodents would elevate systemic fluorine levels through *in vivo* biotransformation and that any resulting dental effects would reflect the combined influence of fluoride, the parent compound, and its primary metabolites (7:3-FTCA and PFOA) (Figure 1B). We investigated the effects of chronic oral exposure to 8:2 FTOH for 90 days^46^ on dental development in male and female C57BL/6J mice (Figure 1C). The doses of 8:2 FTOH used in this study were selected based on prior toxicological research in rodent oral gavage models.^43–46^ Although inhalation is considered the primary route of human exposure to 8:2 FTOH, humans are also exposed through ingestion, including house dust and dietary sources.^27–29^ Rodent toxicokinetic studies show that 8:2 FTOH is rapidly absorbed and cleared after oral dosing, with 22–41% oral bioavailability in rats.^31^ Because no human TK or biomonitoring data exist for parent 8:2 FTOH, differences between inhalation and oral absorption in humans remain unknown. Therefore, we used oral gavage as a controlled and reproducible method to ensure precise dose delivery for toxicological evaluation.

Rodent incisors, particularly those of mice and rats, are widely recognized as a robust model for investigating dental development and pathology.^47^ Their structural and developmental similarities to human enamel make them particularly suitable for evaluating environmental impacts on tooth formation. Rodent incisors grow throughout life, thus allowing researchers to observe all stages of enamel and dentin formation within a single incisor. This dynamic growth makes them especially valuable for studying the effects of systemic exposures on odontogenesis over time.^48^ Given these advantages, the rodent incisor model provides a sensitive and physiologically relevant system for assessing how environmental toxicants such as 8:2 FTOH affect dental development and thus has implications for pediatric dental health and environmental risk assessment.

In this study, we observed a distinct pattern of dental abnormalities—including enamel and dentin hypoplasia, altered mineral density, and microstructural disorganization—accompanying increases in fluoride, PFOA, and 7:3-FTCA. Notably, these defects diverge from classical dental fluorosis, suggesting that 8:2 FTOH—and other PFAS capable of similar metabolic conversion - may represent an understudied source of fluorine accumulation and a potential, previously unrecognized contributor to cryptogenic odontogenic abnormalities.

## 2 Materials and Methods

### 2.1 Reagents

8:2 FTOH (Cat. H084525G, Tokyo Chemical Industry, Tokyo, Japan), with a manufacturer-reported purity of >98%, chemically identified as 1H,1H,2H,2H-heptadecafluoro-1-decanol, was used in this study. Solutions of 8:2 FTOH were freshly prepared 1 day prior to oral gavage at the indicated concentrations using the vehicle, 0.5% carboxymethylcellulose, an inert aqueous vehicle that does not promote abiotic degradation (Sigma-Aldrich, Saint Luis, MO, USA). Our 24 h vehicle checks are consistent with this: no fluoride was detected in 24-h aged 8:2 FTOH dosing solutions in 0.5% CMC, as confirmed by our stability check (Supplementary Table 1). The dosing solutions were not analytically confirmed before administration. Plastic feeding tubes (Cat.FTO-20–38, INSTECH, Plymouth Meeting, PA, US) were used for oral gavage.

### 2.2 Animals

This study was conducted in accordance with the ARRIVE guidelines 2.0, and the completed author checklist is provided as Supporting Information. All animal procedures were performed in compliance with the institutional guidelines for the use of vertebrate animals. The animal protocol was approved by the Institutional Animal Care and Use Committees (IACUC) of Nova Southeastern University (Protocol No. 2023.02.MSuz1), which is accredited by the Association for Assessment and Accreditation of Laboratory Animal Care International (AAALAC). Proof of ethical approval is available upon request.

A schematic representation of the experimental design is shown in Figure 1C. 8:2 FTOH was administered to mice daily by oral gavage for 90 days based on a previously established protocol.^46^ Mice (C57BL/6J) were obtained from The Jackson Laboratory (Bar Harbor, ME, USA). Five-week-old male and female mice were acclimated in-house for 1 week before being randomly assigned by block randomization to one of three groups (N = 6/group males, N = 7/group females): Vehicle control (0 mg/kg body weight/day), low dose (50 mg/kg body weight/day), or high dose (125 mg/kg body weight/day) of 8:2 FTOH. The 8:2 FTOH doses used in this study were selected based on prior toxicological research in animal models.^43–46^ In our preliminary study (an unpublished pilot study conducted in our laboratory), the selected 8:2 FTOH doses produced secondary plasma PFOA concentrations within the upper range of serum PFOA levels reported in occupational cohorts highly exposed to PFOA (tens to thousands of ng/mL).^49–52^ These levels were used as an internal-dose anchor, meaning that comparisons were based on achieved PFOA concentrations rather than an exposure-type equivalence (e.g., exposure dose or exposure route). This internal dose alignment supports the translational relevance of our model for high-exposure scenarios, enabling investigation of biological effects associated with elevated PFAS body burdens.

The experimental unit was the individual animal, and the total number of animals was 39. The sample size was determined based on prior studies and ethical considerations to minimize animal use while ensuring sufficient statistical power. During the 13-week experimental period, the mice received daily oral gavage of 8:2 FTOH at approximately the same time each day. Food (PicoLab Verified 75 IF, LabDiet, St. Louis, MO, USA) and distilled water (supplied from the central system) were provided ad libitum. Body weight was recorded weekly throughout the exposure period. No statistically significant differences in body weight changes were observed among the treatment groups of the same sex and age (Supplementary Figure S1).

At the end of the 13-week treatment, the animals were euthanized 24–36 h after the final 8:2 FTOH treatment to collect tissue samples. Mandibular and maxillary incisors were collected and subjected to the following analyses: quantitative light-induced fluorescence (QLF), Vickers microhardness testing, microcomputed tomography (micro-CT), scanning electron microscopy (SEM), SEM energy-dispersive X-ray spectroscopy (SEM-EDX), and histological examination. Urine, plasma, and femur were collected to quantify fluoride concentrations. PFOA and PFOS concentrations in plasma were measured. To minimize bias, investigators performing outcome assessments and data analysis were blinded to group allocation.

### 2.3 Photography of Mouse Incisors

Following sacrifice, photographs of the maxillary and mandibular incisors were taken using a Nikon D7500 digital camera equipped with an AF-S DX Micro NIKKOR 85 mm f/3.5G ED VR lens (Nikon, Tokyo, Japan). Representative images of male and female mice from each treatment group are shown. To evaluate unerupted regions of the maxillary incisors, each tooth was carefully extracted up to the apical end from the alveolar bone, embedded in wax, and photographed.

### 2.4 Quantitative Light-Induced Fluorescence Assay (QLF)

QLF has been previously employed to objectively assess enamel defects such as dental fluorosis in mice.^48^ Mandibular incisors were dissected in pairs and analyzed using a Nikon epifluorescence microcamera (Nikon, Tokyo, Japan) equipped with a Chroma Gold 11006v2 filter cube (excitation: D360/40x; dichroic mirror: 400DCLP; emission: E515LPv2). The resulting fluorescence images were converted to 8-bit grayscale and analyzed using the ImageJ. software (http://imagej.net/ij/). For each animal, fluorescence intensity on the facial (outer) enamel surface was determined by averaging measurements taken from 10 predefined sites on both incisors.

### 2.5 Micro-CT Analysis

Postsacrifice, three-dimensional imaging of the mandibular incisors and surrounding jawbone was performed using microcomputed tomography (micro-CT). Scans were obtained using a Skyscan 1176 micro-CT scanner (Bruker, Billerica, MA, USA) at 50 kV and 500 μA, with a slice thickness of approximately 9 μm/voxel. Image reconstruction was performed using the NRecon software, and enamel and dentin mineral density were quantified using the CTAn software (Bruker), referencing calibrated mineral density phantoms. Enamel and dentin thickness were defined at the mesial root level of the first molar using frontal cross section images.

### 2.6 Vickers Microhardness Testing of Mouse Incisor Enamel

Vickers microhardness testing was performed as previously described.^53^ Briefly, after micro-CT analysis, mandibular incisors were gently separated from the jawbone using a scalpel and embedded in epoxy resin (Epofix cold-setting embedding resin; Electron Microscopy Sciences, Hatfield, PA, USA). The resin blocks were roughly polished to the midpoint sagittally, and the surfaces were polished with diamond abrasive sheets (grain size: 0.3–30 μm; Maruto Instruments, Tokyo, Japan) to obtain a mirror-like surface. Microhardness indentation was conducted using a Vickers microhardness tester (ALPHA-MHT-1000Z, PACE Technologies, Tucson, AZ, USA) equipped with a square-based pyramid diamond indenter with a 25 g load applied for 10 s. Measurements were taken at each 8 points in the tip and middle regions of incisors ranging 4–5 mm from the tip (Supplementary Figure S2A) and in the midenamel and the mid-dentin layers (Supplementary Figure S2C).^53^ Eight indentations per enamel and dentin in the region were performed. The mean Vickers hardness value (HV) was calculated for each mouse.

### 2.7 SEM, SEM-EDX, and Elemental Mapping Analysis of Enamel and Dentin

Resin-embedded mandibular incisors were analyzed using SEM and SEM-EDX, following previously described.^42^ SEM images of the middle dentin were acquired as backscattered electron (BSE) images using a Quanta 200 SEM (FEI Company, Hillsboro, OR, USA). Elemental mapping was conducted using the Xplore 30 EDS system and the Aztec Live software (Oxford Instruments, Abingdon, UK) to visualize the distribution of elements in the middle of enamel and dentin. The elements analyzed included calcium (Ca), phosphorus (P), sodium (Na), magnesium (Mg), carbon (C), oxygen (O), and strontium (Sr). After EDX analysis, enamel surfaces were etched with 37% phosphoric acid for 40 s, dried thoroughly, gold-coated, and examined by SEM to evaluate enamel ultrastructural features in the tip and middle regions.

### 2.8 Histological Analysis

Maxillary incisors were collected after 90 days of exposure to 8:2 FTOH and fixed in 4% paraformaldehyde for 24 h, followed by decalcification in 10% EDTA for 3 weeks. The tissues were then embedded in paraffin, and 5 μm sagittal sections were prepared. Hematoxylin–eosin (H&E) staining was performed using Harris hematoxylin and Eosin-Y Solution (Thermo Fisher Scientific, Waltham, MA, USA). For immunohistochemistry (IHC), deparaffinized sections were hydrated and heated in 0.01 M citrate buffer at 60 °C overnight to retrieve antigenicity.

Sections were incubated with rabbit anti-KLK4 antibody (1:500; Abcam, Waltham, MA, USA), rabbit anti-Amelogenin antibody (1:100; Abcam) or with rabbit monoclonal IgG XP isotype negative control antibody (1:100; Cell Signaling Technology, Danvers, MA, USA). A secondary antibody conjugated with horseradish peroxidase was applied using the Vectastain Elite ABC-HRP Kit (PK-6101, Vector Laboratories, Newark, CA, USA), followed by visualization with the ImmPACT VIP substrate (SK-4605, Vector Laboratories). Counterstaining was performed with methyl green for 4 min at 60 °C. Sections were examined under light microscopy (Revolve, ECHO, San Diego, CA, USA). At least three animals per group, or all animals meeting predefined quality criteria (e.g., intact, high-quality tissue samples without technical artifacts), were included in the histological analysis, and representative images are presented.

### 2.9 Fluoride Measurements in Plasma, Urine, and Bones

After 8:2 FTOH exposure for 90 days, we collected plasma, urine, and femurs from all animals. Urine and plasma were stored at −20 °C, and femurs were stored in 70% ethanol at 4 °C. Before fluoride measurement, plasma and urine were thawed. Bone samples were dried overnight at 60 °C, ashed in alumina crucibles at 600 °C for 5 h with a 150 °C/h ramp rate (Thermolyne Furnace, FB1315M, Thermo Fisher Scientific), and pulverized using a spatula.

Urine: 20–50 μL of urine was diluted 1:1 with ultrapure water and then 1:1 with TISAB to adjust the pH (5–6), maintain the ionic strength,^54^ and reach a volume of ≥75 μL. The samples were placed between a Petri dish and a double-channel fluoride ion-selective electrode (Orion 9609BNWP, Thermo Fisher Scientific) connected to an ion concentration meter (Orion Dual Star pH/ISE Meter, Thermo Fisher Scientific). Due to low urine volumes collected from individual female mice, urine samples from two duos and one trio of female mice within each treatment group were pooled prior to analysis. This pooling approach ensured adequate sample volume for fluoride quantification using ion-selective electrode methods, which require a minimum sample volume of 20 μL.

Plasma and bone: Diffusion samples were prepared as previously described.^55–58^ Briefly, 75 μL of plasma or 5.0–5.8 mg of bone ash was added to 3 mL of ultrapure water in the bottom of a 60 mm × 15 mm Petri dish. A 50 μL 0.05 N NaOH trap was placed onto the inside surface of the lid. The lid was sealed to the dish with petrolatum, and 3 mL hexamethyldisiloxane (HMDS, Thermo Fisher Scientific)-saturated 3 N sulfuric acid solution was injected through a hole, which was sealed immediately. Acid digestion and HMDS-facilitated diffusion occurred for 4–5 h (plasma) or 16 h (ash) at room temperature on a rocker. Afterward, the lid was inverted and 15–20 μL of 0.2 N acetic acid was added to the trap to adjust pH to 5. The solution was aspirated and adjusted to 75 μL with ultrapure water. Fluoride was measured as described above.

Calibration and Detection Limits: Fluoride standards were prepared in parallel with all samples. Concentrations <0.02 ppm were below ISE detection and recorded as “not detected”.

### 2.10 Plasma PFOA and 7:3 FTCA Measurements

Plasma was collected after 90 days of exposure to 8:2 FTOH. Plasma samples were sent to Eurofins (West Sacramento, CA, USA) to measure 8:2 FTOH-derived PFOA and 7:3 FTCA levels. Quantification was performed using liquid chromatography–mass spectrometry (LC-MS) with validated analytical protocols and appropriate calibration standards by Eurofins. All analyzed samples showed PFOA and 7:3 FTCA concentrations above the reporting limit (RL) (150 and 20 ppb, respectively), as defined by Eurofins. Owing to budgetary constraints, three representative plasma samples per group were randomly selected for analysis of the PFOA and 7:3 FTCA levels. Randomization was performed using Microsoft Excel by generating random numbers and sorting the samples accordingly to ensure unbiased selection.

### 2.11 Statistical Analysis

For each sex, the effects of the 8:2 FTOH dose were analyzed using one-way ANOVA followed by Dunnett’s multiple comparisons test for post hoc analysis. To assess the differential dose effects between sexes, two-way ANOVA was used, followed by Tukey’s multiple comparisons test. All statistical analyses were performed using GraphPad Prism version 10 (GraphPad Software, Boston, MA, USA). Statistical significance was defined as *p* < 0.05.

## 3 Results

### 3.1 8:2 FTOH Exposure Increased Plasma Levels of PFOA and 7:3 FTCA

Following 90 days of systemic 8:2 FTOH treatment, plasma was collected 24 to 36 h after the last 8:2 FTOH administration. Plasma levels of PFOA and 7:3 FTCA, two main 8:2 FTOH metabolites, were quantified by LC/MS. PFOA and 7:3 FTCA showed a significant increase in plasma in both sexes (Figure 2 and Table 1). In contrast, PFOS, which is unrelated to 8:2 FTOH metabolism,^59^ showed no changes in plasma levels following 8:2 FTOH administration in either sex (Supplementary Figure S3). Notably, in the high-dose group, male animals displayed higher plasma PFOA and 7:3 FTCA concentrations compared to females (Supplemental Figure S3).

**Figure 2:**
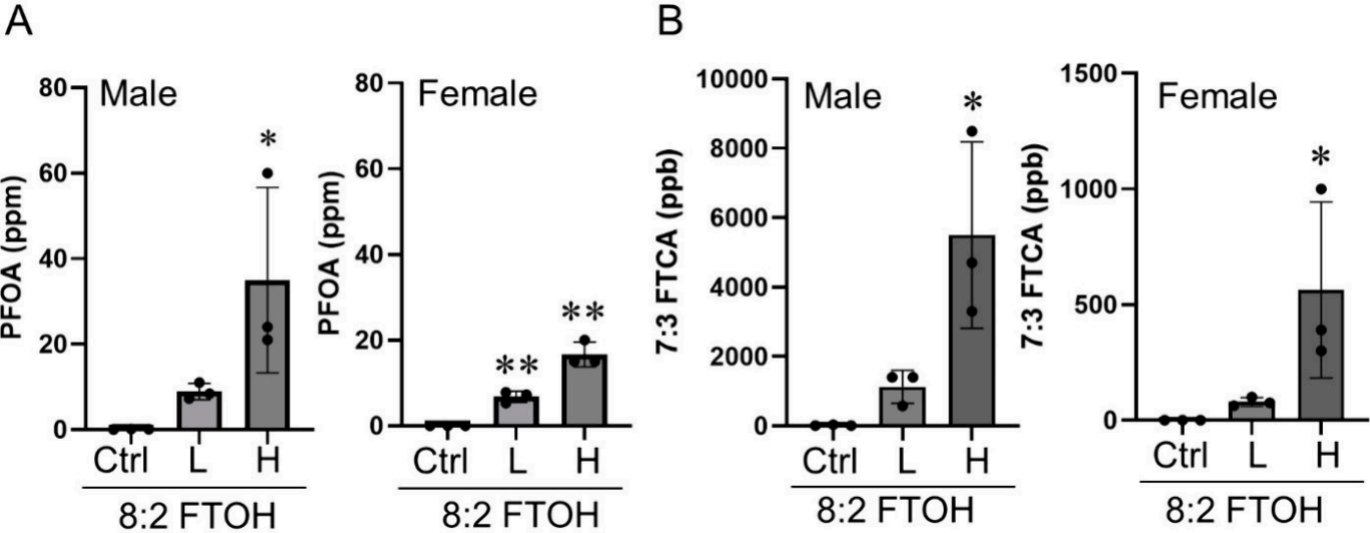
Increase in plasma PFOA and 7:3 FTCA levels after 8:2 FTOH exposure. After 8:2 FTOH treatment for 90 days, plasma levels of PFOA (A) and 7:3 FTCA (B) were analyzed. Plasma PFOA and 7:3 FTCA levels were significantly increased by the 8:2 FTOH treatment compared to the control in both sexes. Statistical analysis was performed in each sex using one-way ANOVA followed by post hoc analysis. Data are presented as the mean ± standard deviation (SD). *N* = 3/group. *p < 0.05, **p < 0.01 significant differences. Ctrl: control, L: low dose, H: high dose.

**Table 1:** Systemic Accumulation of PFOA, 7:3 FTCA, and Fluoride in Mice Following 8:2 FTOH Exposure^a^

		PFOA (ppm)	7:3 FTCA (ppb)	fluoride (ppm)		
8:2 FTOH	Sex	Plasma	Plasma	Urine	Plasma	Bone
0 (mg/kg)	Male	0.095 ± 0.064	22.67 ± 15.04	5.200 ± 1.936	0.022 ± 0.007	897.8 ± 92.29
	Female	0.002 ± 0.001	0.083 ± 0.072	4.303 ± 1.479	0.017 ± 0.005	890.8 ± 109.5
50 (mg/kg)	Male	8.900 ± 1.900	1127 ± 473.4	7.183 ± 1.656	0.070 ± 0.032	1421 ± 196.1
	Female	6.800 ± 1.323^b^	79.67 ± 18.77	6.300 ± 1.914	0.030 ± 0.008	1274 ± 207.7
125 (mg/kg)	Male	35.00 ± 21.70^b^	5500 ± 2691^b^	17.820 ± 3.304^c^	0.633 ± 0.603^b^	3558 ± 827.8^c^
	Female	6.67 ± 2.887^c^	563.3 ± 380.8^b^	19.070 ± 10.09^b^	0.077 ± 0.048^c^	3796 ± 1189^c^

a After 8:2 FTOH treatment for 90 days, the PFOA, 7:3 FTCA and fluoride levels in urine, plasma, and bone were measured. In both sexes, PFOA, 7:3 FTCA, and fluoride levels significantly increased in each tissue in the high-dose 8:2 FTOH group

b *P* < 0.05.

c *P* < 0.01 vs 0 (mg/kg) in each sex.

### 3.2 Fluoride Accumulation in Urine, Plasma, and Bone Following 8:2 FTOH Exposure

Fluoride levels in urine, plasma, and bone (femur) increased in a concentration-dependent manner following 90 days of 8:2 FTOH administration. Notably, high-dose exposure resulted in a significant elevation of fluoride accumulation, ranging from 17 to 19 ppm in urine, 0.08 to 0.6 ppm in plasma, and 3558 to 3795 ppm in bone compared to the control groups. These increased fluoride levels are comparable to those observed in murine dental fluorosis models treated with 50–100 ppm fluoride.^41,60^ This is a consistent fluoride accumulation trend across both sexes (Figure 3 and Table 1). When assessing sex-specific differences, fluoride levels in plasma were higher in males than in females, whereas fluoride levels in urine or bone did not differ significantly (Supplemental Figure S4). These results suggest that FTOHs or other PFAS can represent indirect fluoride sources via biotransformation, which can cause adverse health effects caused by fluoride bioaccumulation.

**Figure 3:**
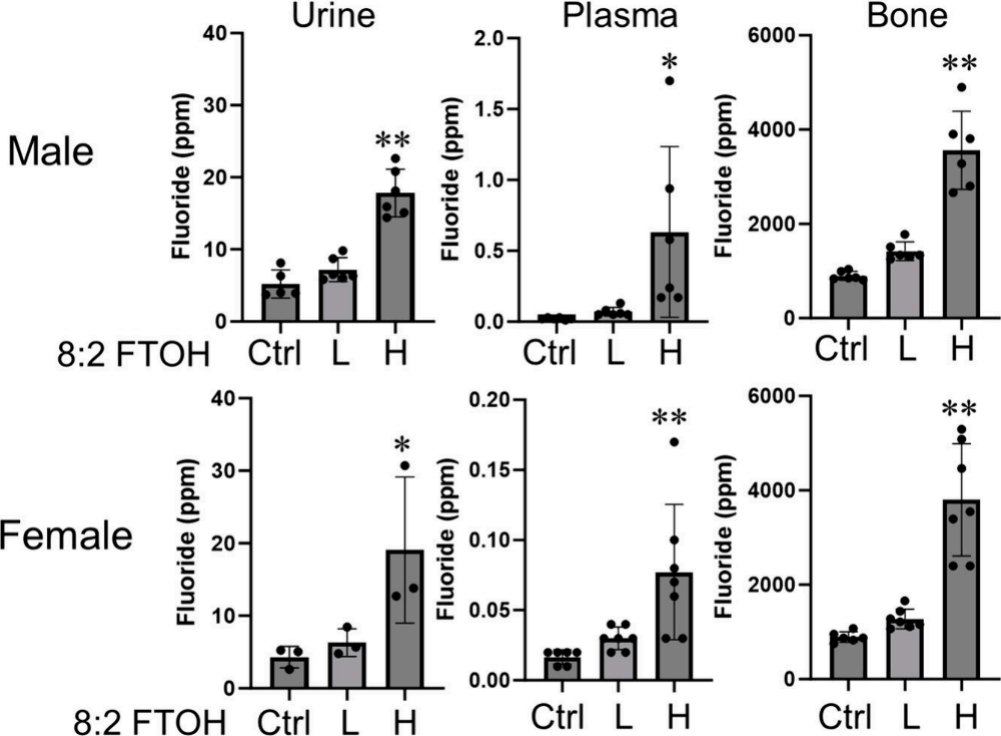
Increase of fluoride in urine, plasma, and bone after 8:2 FTOH exposure. After 8:2 FTOH treatment for 90 days, fluoride levels in urine, plasma, and bone were measured. In both sexes, the fluoride levels in each tissue significantly increased in the high-dose 8:2 FTOH group. Statistical analysis was performed in each sex using one-way ANOVA followed by post hoc analysis. Data are presented as the mean ± standard deviation (SD). *N* = 6–7/group. Three urine samples from two to three female mice per treatment group were pooled for analysis (*N* = 3 pooled samples/group). *p < 0.05, **p < 0.01 L: low dose, H: high dose.

### 3.3 Chalky Enamel and Reduced Iron Content Indicate Hypoplasia after High-Dose 8:2 FTOH Treatment

To assess the impact of 90-day systemic 8:2 FTOH administration on tooth development, optical evaluation of mouse incisors was performed. Representative images (Figure 4A) show whitening and patchy opacities of mandibular incisors (arrows) in males and females at the high-dose of 8:2 FTOH. The doses used in this study were based on prior animal toxicology research and resulted in plasma PFOA levels in mice comparable to those seen in occupationally exposed humans. This supports the relevance of the dosing model for studying health effects linked to high PFAS exposure. To examine unerupted regions, maxillary incisors were carefully dissected from the maxilla and mounted in wax (Figure 4B). The unerupted portion (corresponding to the maturation stage of enamel development) constitutes a substantial proportion of the total maxillary incisor length compared with the erupted portion. After 8:2 FTOH exposure, unerupted enamel displayed a chalky texture and white appearance (Figure 4B, arrow heads), thus indicating enamel hypoplasia. QLF is commonly applied for objective detection of enamel hypoplastic lesions (e.g., chalky discoloration, demineralization, and fluorosis).^48^ QLF values were significantly increased in a dose-dependent manner, with no sex differences. This indicates that enamel hypoplasia is caused by an increase of fluoride following 8:2 FTOH exposure in both males and females (Figure 4C and Supplemental Figure S5). Micro X-ray fluorescence (micro-XRF) analysis of sagittal sections (Supplemental Figure S6) revealed reduced enamel integrity and decreased iron (Fe) content in the high-dose group, further supporting structural compromise.

**Figure 4:**
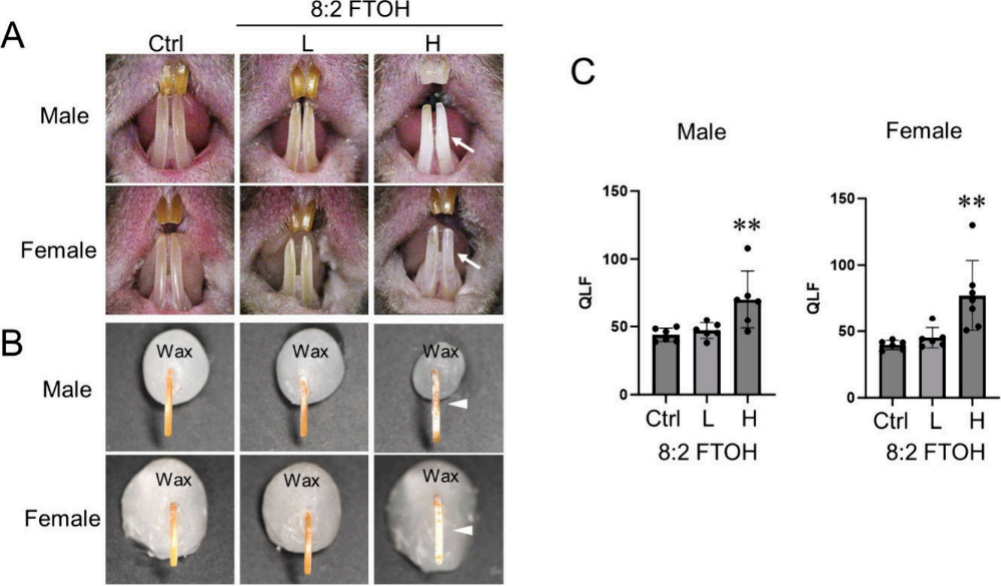
Optical tooth morphological changes with enamel hypoplasia caused by 8:2 FTOH exposure. (A) Representative images of incisors after 90 days of 8:2 FTOH administration. In both male and female mice, high-dose 8:2 FTOH exposure resulted in enamel hypoplasia, manifesting as chalky white enamel (arrows). (B) Representative images of maxillary incisors dissected from the jawbone. Incisors were mounted on wax. Enamel hypoplasia lesions were observed in the unerupted part of the tooth (arrow heads). (C) QLF, which reflects the enamel hypoplasia level, was analyzed in each sex using one-way ANOVA followed by post hoc analysis. High concentrations of 8:2 FTOH significantly increased enamel hypoplasia in both sexes (**p < 0.01). A linear regression trend analysis with dose treated as a continuous variable demonstrated a significant positive dose–response relationship (Male: β = 0.61, *p* < 0.001, Female: β = 0.223, *p* < 0.001). Data are presented as the mean ± standard deviation (SD). *N* = 6–7/group, reflecting exclusion of damaged samples from the originally planned group sizes. L: low dose, H: high dose.

### 3.4 Systemic 8:2 FTOH Exposure Compromises Enamel and Dentin Mechanical Integrity

Mechanical integrity is critical for the function of mineralized tissues. To evaluate the effects of 8:2 FTOH exposure, enamel and dentin microhardness were assessed using the Vickers microhardness test. Indentations were performed at the enamel tip, midenamel, and mid-dentin regions (Supplemental Figure S2). High-dose 8:2 FTOH exposure reduced microhardness values in both enamel and dentin (Figure 5). In males, enamel on the tip (erupted enamel) showed a trend toward lower microhardness values compared with that in the controls (Figure 5A), while in females, the microhardness value at the same site was significantly lower compared with that in controls (Figure 5B). Microhardness in the middle enamel (unerupted enamel) was significantly decreased by high-dose 8:2 FTOH in both males and females. Dentin microhardness was reduced in both sexes at high doses, with females showing sensitivity even at low doses (Figure 5B). This is evident from the dose and sex interaction of *p* = 0.0339 (Supplementary Figure S7). Overall, enamel microhardness declined with increasing 8:2 FTOH exposure across sexes, without significant sex differences. In contrast, dentin microhardness showed sex differences, which may have been partly due to baseline variations (Supplemental Figure S5).

**Figure 5:**
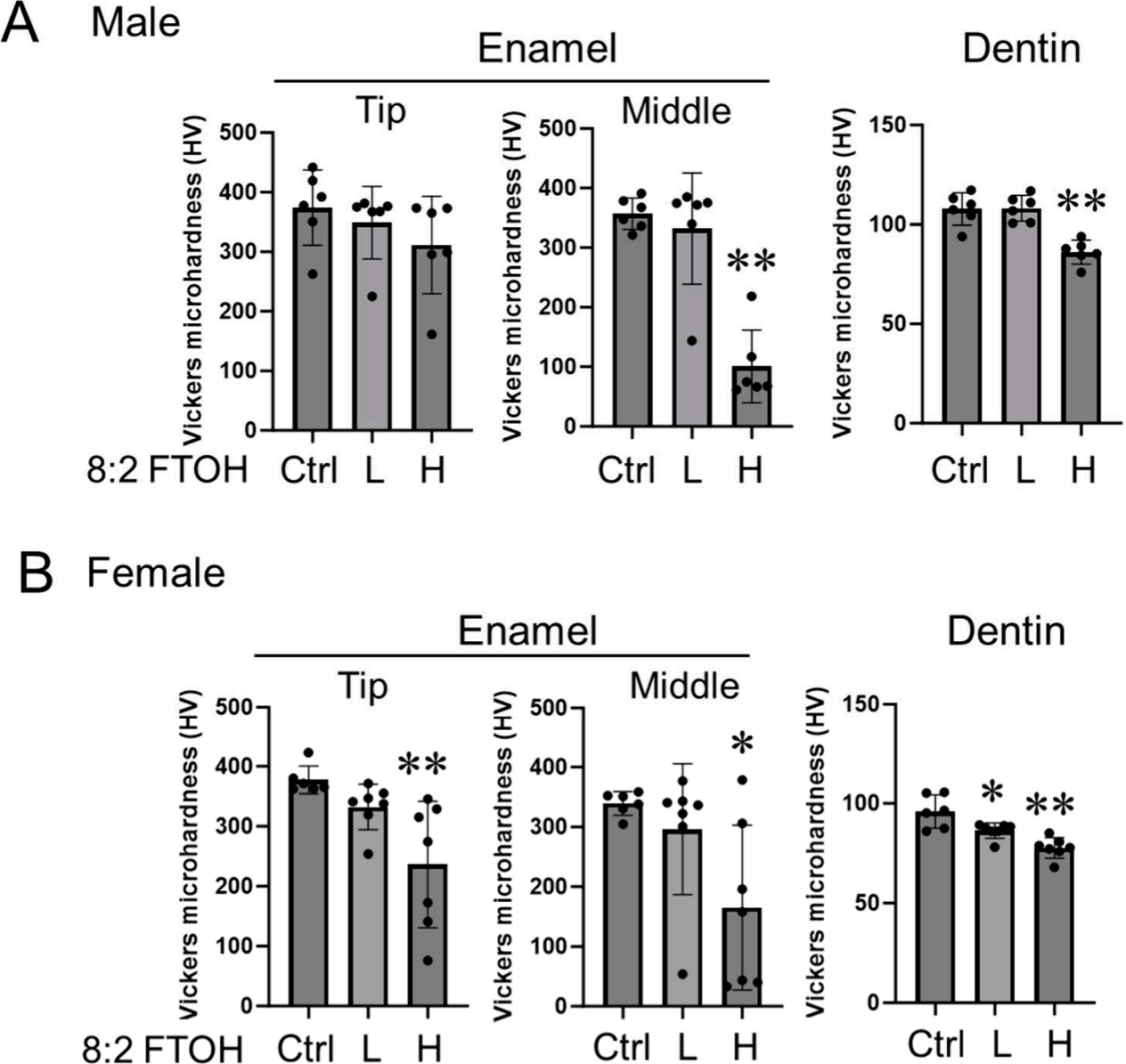
Suppression of the enamel and dentin mechanical properties by 8:2 FTOH exposure. Mechanical properties of enamel and dentin were analyzed by Vickers microhardness testing. Enamel was analyzed in the tip (corresponding to the erupted enamel) and in the middle regions (corresponding to the maturation stage enamel), and dentin was evaluated in the middle region. Vickers microhardness in enamel and dentin was suppressed by 8:2 FTOH in male (A) and female (B). Statistical analysis was performed in each sex using one-way ANOVA followed by post hoc analysis. Data are presented as the mean ± standard deviation (SD). *p < 0.05, **p < 0.01. *N* = 6–7/group, reflecting exclusion of damaged samples from the originally planned group sizes. L: low dose, H: high dose.

### 3.5 Three-Dimensional (3D) Reconstruction Highlights Structural and Mineralization Defects in Enamel and Dentin Induced by 8:2 FTOH

Representative 3D-reconstructed sagittal mandibular incisor images from both sexes are shown in Figure 6A. High-dose 8:2 FTOH exposure disrupted the enamel architecture, revealing partial structural defects and reduced mineral density (Figure 6A, arrowheads), which are consistent with reduced microhardness of enamel and dentin and suggest impaired tooth maturation. The pulp tissue appeared enlarged, consistent with dentin hypoplasia. Quantitative analysis of enamel and dentin volume, thickness, and mineralization showed a dose-dependent decline that was most pronounced in the high-dose group (Figure 6B). Evaluation of sex differences did not identify significant differences in enamel volume, thickness, or mineralization (Supplemental Figure S8). In contrast, dentin thickness and mineralization were lower in females than in males following high-dose 8:2 FTOH exposure. These findings suggest that female dentin may be more susceptible to the effects of 8:2 FTOH, as shown by the dentin thickness *p* = 0.0145 and mineral density *p* = 0.0275 (Supplemental Figure S8). These findings are consistent with the observed differences in mechanical properties (Supplemental Figure S7).

**Figure 6:**
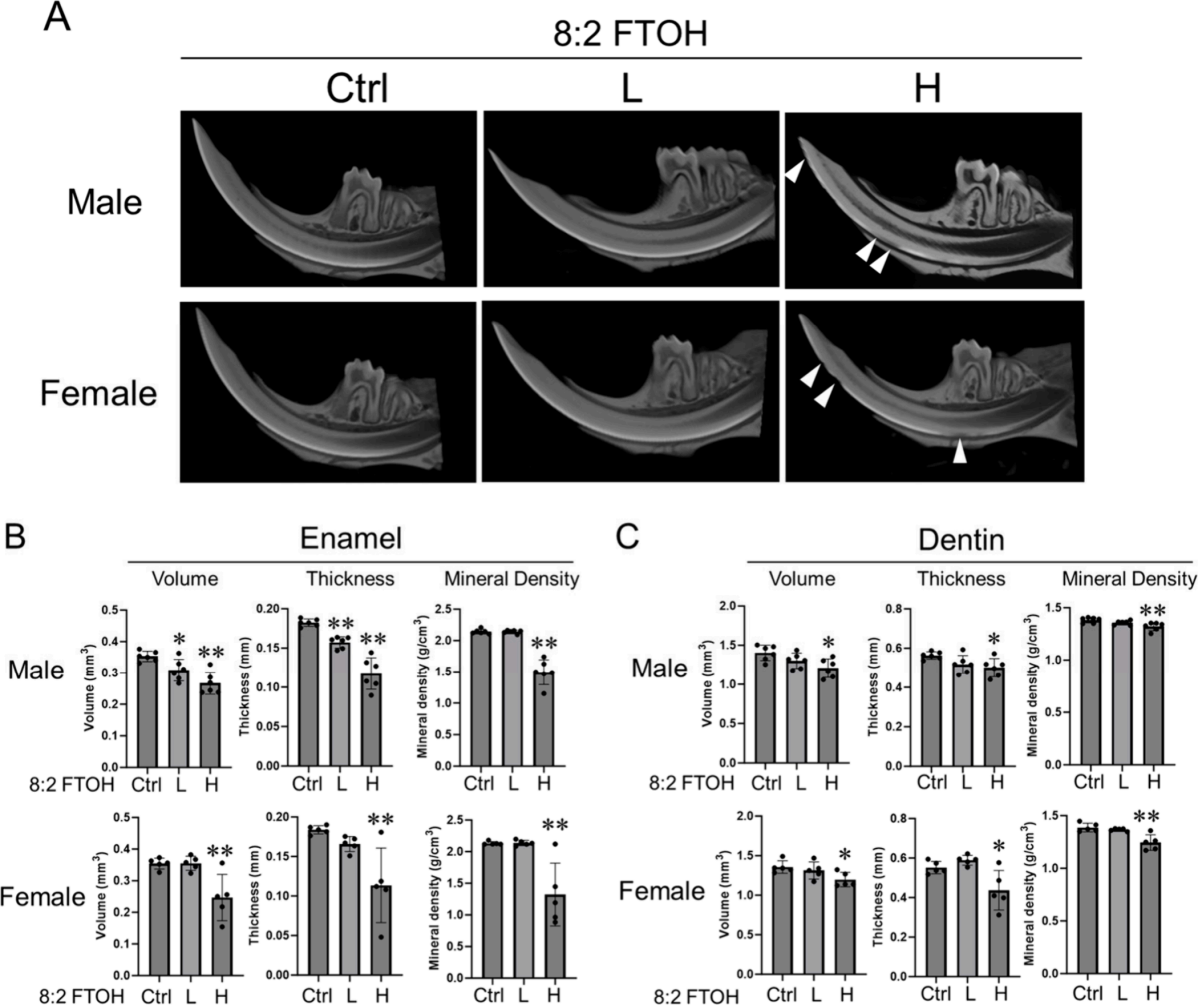
Three-dimensional analysis of enamel and dentin affected by 8:2 FTOH exposure. (A) Representative micro-CT images of a mandible sagittal section with the incisor treated with 8:2 FTOH. High-dose 8:2 FTOH caused enamel loss or hypo-mineralized lesions (arrowhead). (B, C) Three-dimensional evaluation of volume, thickness, and mineral density in enamel (B) and dentin (C) was performed in each sex. Suppression of volume, thickness, and mineral density in enamel and dentin was observed in both sexes. Statistical analysis was performed in each sex using one-way ANOVA followed by post hoc analysis. Data are presented as the mean ± standard deviation (SD). *N* = 6–7/group. *p < 0.05, **p < 0.01. L: low dose, H: high dose.

### 3.6 Disrupted Expression of Amelogenin and KLK4 Indicates Functional Hypoplasia in Enamel Formation after 8:2 FTOH exposure

Incisors from the control and high-dose 8:2 FTOH-treated mice were examined histologically after 90 days of systemic exposure. H&E staining revealed no apparent morphological changes in ameloblasts following 8:2 FTOH exposure during the secretory stage (SEC) compared with the control (Figure 7A, 7B). However, despite their normal appearance, immunohistochemical analysis showed reduced expression of amelogenin (a key marker of the secretory stage) in ameloblasts in the FTOH-treated group (Supplemental Figure S9A), suggesting functional impairment despite preserved morphology during the SEC.

**Figure 7:**
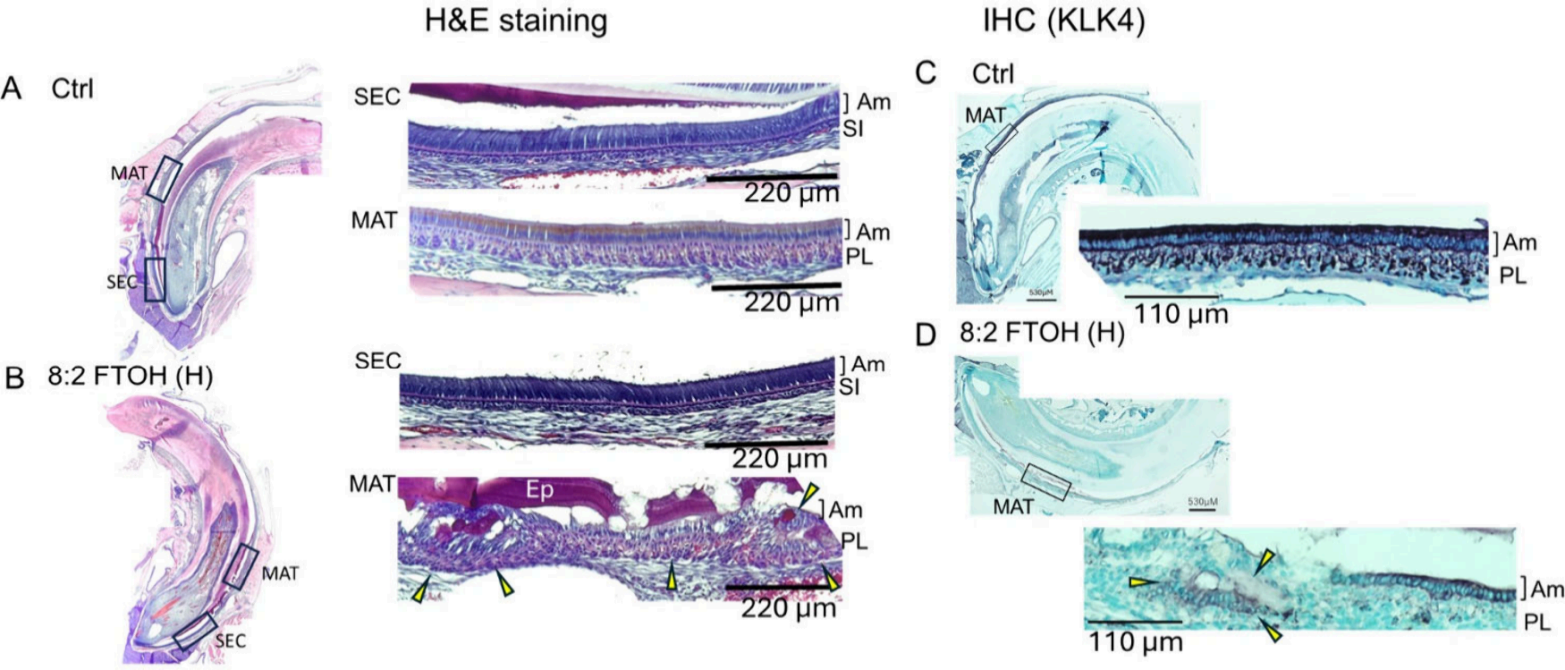
Histological evaluation of ameloblasts affected by 8:2 FTOH exposure. (A and B) Representative hematoxylin and eosin (H&E) staining images of maxillary incisors treated with 8:2 FTOH (A: control and B: high dose). Magnified images (right panels) of the boxed area of the secretory stage (SEC) and maturation stage (MAT) are shown. Compared to the control, 8:2 FTOH disrupted the ameloblast layer (B, arrowheads), and aberrant enamel protein (Ep) remained in MAT. (C and D) Representative images of Immunohistochemical staining (IHC) for KLK4. Magnified images of the squared area of the MAT show that suppression of KLK4 expression by 8:2 FTOH is pronounced in disrupted ameloblasts (D, arrowheads). Am: ameloblast, Ep: enamel protein, SI: stratum Intermedium, PL: Papillary layer. Scale bars: 220 μM (inset images of A and B); 110 μM (inset images of C and D). H: high dose.

In contrast, during the maturation stage (MAT), ameloblasts exhibited disrupted cellular continuity and disorganized alignment (Figure 5B, MAT, arrowheads). KLK4, a representative marker of maturation-stage ameloblasts, was strongly expressed in a well-organized ameloblast layer in controls (Figure 7C). In the 8:2 FTOH-treated group, KLK4 expression was markedly reduced and accompanied by a loss of cellular organization (Figure 7D, arrowheads). The specificity of amelogenein and KLK4 immunostaining was confirmed using isotype-matched IgG negative controls, which showed no detectable staining (Supplemental Figure 9B).

### 3.7 Disruption of Enamel Prism Architecture and Dentin Tubule Integrity Following 8:2 FTOH Exposure

Mandibular incisors were resin-embedded, mirror-polished, etched with phosphoric acid, and gold-coated prior to SEM analysis. Microstructural evaluation was performed at the tip and middle regions of the enamel, corresponding to the sites used for microhardness testing. Representative SEM images from the control and high-dose 8:2 FTOH groups are shown in Figure 8, with low-magnification images presented in the upper row and high-magnification images in the lower row. In the controls, enamel exhibited a ribbon-like pattern formed by the regular arrangement of intertwined enamel prisms in the middle region (Figure 8C) and at the tip (Figure 8D). In contrast, the 8:2 FTOH-treated group retained the presence of prisms but lacked the organized ribbon-like architecture, with the structural disruption more pronounced in the middle region (Figure 8G) than at the tip (Figure 8H).

**Figure 8:**
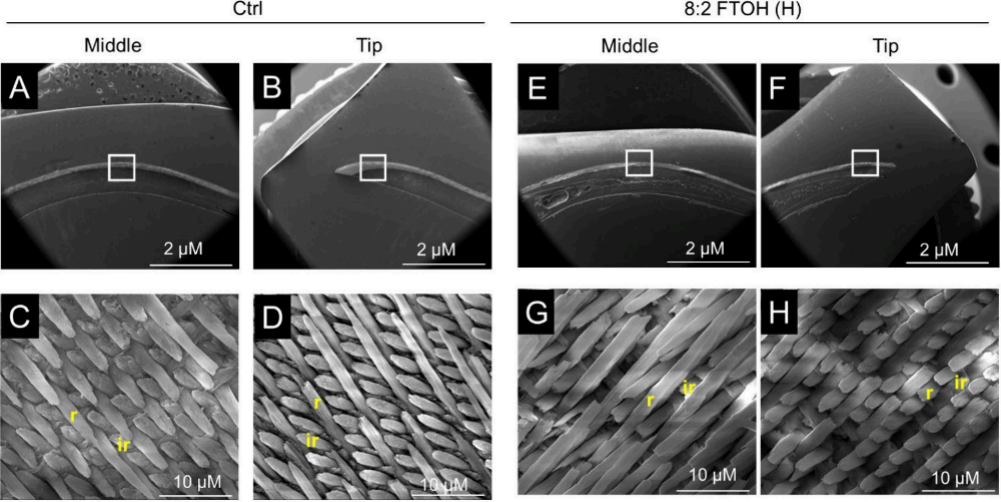
Affected enamel prism structure caused by 8:2 FTOH exposure. Representative SEM images of the enamel prism in the middle and tip regions of the mandibular incisor. Magnified images (lower panels) of the boxed area in each region are shown. (A–D) Vehicle control. The enamel prism structure shows a regular arrangement in both the middle (A, C) and the tip regions (B, D) of the enamel. (E–H) 8:2 FTOH (high dose). Enamel prismatic structures are severely disordered in the middle area (G), showing an irregular rod and inter-rod pattern compared to the tip (H). r: Rod, ir: Inter rod. Scale bars: Panels A, B, E and F, scale bar = 2.0 μm; for panels C, D, G and H, the scale bar = 10 μm.

The dentin microstructure was examined using backscattered electron imaging of a mirror-polished surface prior to etching and gold-coating. Representative images of the dentin–pulp boundary from all experimental groups and both sexes are presented in Supplemental Figure S10. In the control and low-dose groups, dentin exhibited well-defined tubular structures and a distinct pulp boundary (Supplemental Figure S7A–C). In the high-dose group, granular matrix vesicles, which indicate dentin hypoplasia due to altered odontoblast activity, were observed (Supplemental Figure S10E and F, arrowheads). In addition, structural defects within the dentin (Supplemental Figure S10G and H, arrows) were evident and likely contributed to the observed reductions in microhardness (Figure 5). SEM imaging did not reveal any sex-specific differences in these dentin abnormalities following 8:2 FTOH exposure.

Elemental mapping by SEM-EDX of mirror-polished mandibular specimens focused on the middle regions of enamel and dentin. The results for C, O, F, Na, Mg, P, Ca, Fe, and Sr are shown in weight percent (wt %) (Supplemental Table S2). The carbon content was significantly elevated at the high-dose of 8:2 FTOH in both enamel and dentin. In contrast, magnesium levels were significantly reduced in dentin at the same dose. These findings suggest that enamel and dentin hypoplasia were primarily associated with increased carbon content. The suppression of magnesium, a key element in dentin maturation, may further contribute to the development of dentin hypoplasia.

## 4 Discussion

To our knowledge, this is the first study to comprehensively demonstrate the pathophysiology of PFAS-associated developmental dental anomalies—enamel and dentin hypoplasia—in the context of fluoride accumulation after exposure to PFAS *in vivo*. Our findings suggest that PFAS capable of metabolic conversion (e.g, 8:2 FTOH) may act as underrecognized contributions to fluoride bioaccumulation, potentially helping to explain cryptogenic odontogenic abnormalities.

8:2 FTOH is known to undergo microbial biotransformation in environmental matrices such as activated sludge and soils, where partial defluorination can occur, contributing to environmental sources of PFOA and fluoride ion.^20–24^ Previous studies characterize the toxicokinetics of 8:2 FTOH biotransformation in rodents,^33,36^ yet none have examined fluoride ion production following in vivo exposure to 8:2 FTOH. Consequently, whether mammalian metabolism of 8:2 FTOH can generate fluoride ion remains largely uncharacterized, particularly given the absence of any known mammalian enzyme or physiological pathway capable of directly cleaving the C–F bond. In this study, we hypothesized that chronic 8:2 FTOH exposure in rodents increases systemic fluorine levels through metabolic conversion of the parent compound and its intermediates, and that associated dental effects reflect the combined influence of fluoride, 8:2 FTOH itself, and its primary metabolites, 7:3 FTCA and PFOA, accumulating within mineralized tissues. This framework guided our interpretation of the enamel and dentin abnormalities observed at the high exposure levels.

Excessive systemic fluoride exposure and PFAS exposure have each been independently linked to developmental toxicity, including neurodevelopmental effects as well as dental and skeletal fluorosis.^6–9,61–65^ Our data show that chronic 8:2 FTOH exposure elevates systemic fluoride levels and induces characteristic dental and mineralized tissue defects in vivo. These findings suggest that fluoride generated via PFAS precursor (similar mechanisms of FTOHs) may contribute to PFAS-associated developmental health effects.

Despite these parallels, only limited research has specifically examined the effects of PFAS on dental and craniofacial development. A cohort study showed that prenatal PFAS (perfluorodecanoic acid; PFDA) exposure was associated with shorter palpebral fissure length.^10^ Only a few epidemiological studies have focused on PFAS exposure and dental health, including the association with periodontitis (PFOS and perfluorononanoic acid; PFNA),^11^ dental caries (PFDA),^12^ and developmental defects of enamel (DDE) (PFOS and PFOA).^13,14^ In experimental models, PFAS exposure has been associated with craniofacial deformities in zebrafish (PFOS, PFNA, and PFOA)^15^ and an increased incidence of cleft palate in rodents (PFOS).^16,17^ Although several studies reported tooth malformations in rodents caused by 8:2 FTOH (precursor of PFOA) or other FTOHs (6:2 FTOH, 10:2 FTOH, and their mixtures),^43–46^ these studies did not demonstrate the specific tooth phenotypic, microstructural, and histological characteristics of dental defects caused by FTOHs.

This proof-of-concept study provides initial evidence suggesting that *in vivo* metabolism of 8:2 FTOH may be accompanied by defluorination, as reflected by significant increases in fluoride in plasma, urine, and bone after 8:2 FTOH exposure, alongside dental abnormalities at the highest dose. At present, there is no published evidence identifying a mammalian enzyme or pathway that cleaves the C–F bond of 8:2 FTOH to release inorganic fluoride *in vivo*. While environmental microorganisms can partially defluorinate fluorotelomer compounds,^21–24,66^ such microbial capability does not establish an analogous gut–microbiome–dependent pathway in mammals. Accordingly, we present any microbial contributions *in vivo* as a testable hypothesis. Targeted follow-up should include microbiome-informed designs to determine whether, when, and how fluoride arises during 8:2 FTOH biotransformation *in vivo*, and to delineate any role of the gut microbiome.

The administered doses of 8:2 FTOH (low and high dose) were selected based on prior toxicological studies in rodents.^43–46^ We acknowledge that PFAS toxicokinetics differ substantially between species. Because mice retain PFOA for much longer (half-life ∼ 14 days) compared with rats (hours to a few days depending on sex), dose levels derived from rat studies may result in proportionally higher systemic PFAS burdens in mice than would be expected based on nominal dosing alone. Interpretation of dental and systemic outcomes must therefore rely on measured internal concentrations of PFOA rather than assumptions based on rat-derived dose parameters. Although doses used in animal models^43–46^ exceed typical environmental exposure in the general population, they remain scientifically warranted for modeling high-exposure scenarios. Notably, 8:2 FTOH exposure resulted in high plasma PFOA concentrations in mice, which are comparable to serum levels reported in occupationally exposed individuals, including fluorochemical manufacturing workers with documented concentrations up to 12,000 ng/mL (12 ppm) or higher.^49–52^ While the occupational cohort data primarily reflect direct PFOA exposure,^49–52^ not exposure to 8:2 FTOH, we interpret resulting plasma PFOA levels in mice (within a realistic range in humans) as an internal dose anchor rather than an exposure-type equivalence. This internal dose alignment supports the relevance of our animal model for high-exposure scenarios, enabling investigation of biological effects associated with elevated PFAS body burdens.

Concurrently, the fluoride levels in plasma, urine, and bone were significantly elevated by 8:2 FTOH, and the values were equal to those in the dental fluorosis model in mice treated with 50–100 ppm fluoride.^41,60^ This suggests that 8:2 FTOH exposure is a hidden risk factor for fluoride bioaccumulation, which could primarily contribute to tooth malformation. Although some 8:2 FTOH-mediated tooth anomalies resemble dental fluorosis (increase of QLF, decrease of microhardness, and disrupted ameloblasts), distinct defects were observed in the enamel prism structures between 8:2 FTOH and fluoride exposure.^67^ In addition, the elemental mapping of incisors showed different results between systemic fluoride administration and exposure to 8:2 FTOH, indicating that the two act through distinct mechanisms in inhibiting hard tissue formation. Furthermore, in rodent dental fluorosis models, after dental fluorosis occurs, the removal of fluoride (recovery period) results in some visual recovery of enamel pigmentation in rat incisors.^68^ Meanwhile, a previous study in rats exposed to 8:2 FTOH for 90 days demonstrated that after a three-month recovery period, ameloblastic degeneration/disorganization was still present in male rats previously dosed with 8:2 FTOH at high dose.^46^ These studies suggest that the mechanisms of 8:2 FTOH-mediated tooth malformation may differ from dental fluorosis caused solely by fluoride.

We previously reported that PFOA induces cell death via apoptosis and necroptosis in mouse ameloblast-like cells (ALC) in vitro,^18^ suggesting that elevated PFOA derived from 8:2 FTOH could affect ameloblasts during enamel development *in vivo*. To date, no studies have conclusively demonstrated a direct link between PFOA exposure and tooth malformation. Epidemiological findings remain inconsistent regarding the association between PFOA and dental characteristics. Some studies suggest that PFOA may increase the risk of dental caries prevalence and enamel defects,^12^ including molar-incisor hypomineralization (MIH),^13^ while others report no significant association between PFOA exposure and dental caries prevalence^69^ or a negative association with DDE.^14^ These mixed findings imply that while PFOA, particularly that derived from 8:2 FTOH, may contribute to enamel malformation, fluoride likely plays a more prominent role in enamel malformation.

Our plasma sampling occurred 24–36 h after the final oral gavage, a time frame in which parent 8:2 FTOH would not be expected to remain detectable in mice. This expectation is consistent with the comprehensive mouse toxicokinetic data set from Henderson and Smith (2007),^32^ which reported that 8:2 FTOH was no longer detectable in maternal serum or liver beyond 24 h post-treatment. Given this established rapid clearance, it was reasonable to anticipate that parent 8:2 FTOH would be absent or present only at trace levels at terminal bleed, whereas PFOA—an established, persistent downstream metabolite—would serve as a more reliable internal dose marker at the time dental end points were evaluated. Although prior rodent studies have demonstrated *in vivo* biotransformation of 8:2 FTOH to PFOA and 7:3 FTCA,^31,32,36^ none of these studies reported fluoride release or quantified 7:3 FTCA in mice as downstream products of 8:2 FTOH metabolism. Thus, our measurements of fluoride and 7:3 FTCA at termination represent a novel contribution, providing new insight into potential metabolic outcomes of 8:2 FTOH exposure in mice. In this proof-of-concept study, we focused analytical effort on fluoride and PFOA at the terminal time point, acknowledging that earlier-interval contributions from parent 8:2 FTOH and 7:3 FTCA cannot be excluded. To more fully define exposure dynamics, future studies should incorporate mouse-specific serial sampling across expected half-life windows to quantify fluoride, 8:2 FTOH, 7:3 FTCA, and PFOA, enabling tighter linkage between internal dosimetry and biological outcomes.

Compared to enamel, the effects of PFAS on dentin formation (i.e., dentinogenesis) are even less documented. Odontoblasts and dental pulp stem cells share a lineage with osteoblasts and bone marrow-derived stem cells. Although epidemiological and experimental studies on PFAS-related bone formation have been reported,^70,71^ the impact of PFAS on dentin remains largely unexplored. Addressing this gap highlights the need for further investigation into the effects of PFAS on dentinogenesis. In this study, we provide the first evidence that PFAS exposure influences dentin formation and reveal that 8:2 FTOH exerts distinct effects compared to fluoride alone. Systemic fluoride exposure alters the three-dimensional frontal cross-section of incisors, resulting in abnormal shapes: while control incisors exhibit an oval morphology, fluoride exposure at concentrations of 100 and 125 ppm compresses the incisor into a triangular form^42^ (Supplemental Figure S11A, indicated by arrows). In contrast, exposure to 8:2 FTOH at a high dose resulted in body fluoride levels comparable to those observed with the 100 ppm fluoride treatment but did not induce a triangular shape (Supplemental Figure S11B). This suggests that the pathological mechanisms of 8:2 FTOH differ from those of fluoride. Additionally, fluoride exposure led to external root resorption in the apical region, particularly on the lingual side of teeth (Supplemental Figure S11A, circled areas). Notably, this phenomenon was absent following 8:2 FTOH exposure. Despite the absence of overt morphological changes in the incisor shape or roots, ultrastructural abnormalities in dentin with granular matrix vesicles (Supplemental Figure S8) may compromise tooth integrity. These defects can promote caries development and increase susceptibility to tooth fractures, highlighting the potential life-long dental risks associated with PFAS exposure.

Our findings indicate that the effects of 8:2 FTOH are dose-dependent, with no consistent sex-based differences observed overall. However, some variations in the dentin profiles were observed between sexes, which may reflect baseline physiological differences rather than differential susceptibility. Available experimental evidence indicates that sex-specific differences in responses to fluorotelomer alcohols, including 8:2 FTOH, are not well established. Toxicokinetic studies in rats and mice show that absorption, bioavailability, and clearance of the parent compound 8:2 FTOH are largely similar between males and females, with comparable plasma half-lives and internal dose metrics.^31,36^ In contrast, pronounced sex differences have been consistently observed for the persistent downstream metabolite PFOA formed following 8:2 FTOH exposure, with males exhibiting substantially longer elimination half-lives than females.^31,32^ These published findings are consistent with our results, which show significantly higher plasma PFOA concentrations in males compared with females. In addition, plasma levels of another metabolite, 7:3 FTCA, were approximately 10-fold higher in males than in females. To date, however, there is limited evidence directly linking such sex-dependent toxicokinetic differences to differential health or developmental outcomes specifically attributable to the parent compound 8:2 FTOH. Collectively, these findings highlight a critical data gap and underscore the need for future studies that integrate sex-specific toxicokinetics with apical health outcomes, particularly for PFAS precursors and metabolically active compounds such as 8:2 FTOH.

Following 8:2 FTOH exposure, serum and bone fluoride levels were significantly elevated. These levels fall within ranges associated with dental fluorosis in mouse models. This finding supports an 8:2 FTOH–mediated fluoride contribution to disrupted tooth development. The effect is likely driven by interference with enamel formation and mineralization during odontogenesis. Importantly, several features observed after 8:2 FTOH exposure differ from those typically induced by fluoride alone. This indicates that classical fluorosis mechanisms do not fully explain the findings. The results suggest involvement of additional pathological pathways. These pathways may be mediated by the parent compound 8:2 FTOH and its metabolites, including PFOA and 7:3 FTCA.

A key limitation of this study is that it does not fully disentangle the contributions of 8:2 FTOH, its major metabolites PFOA, 7:3 FTCA, and minor metabolites, and fluoride on dental defects. Although our previous *in vitro* work suggested that PFOA can affect ameloblasts during enamel development.^18^ The isolated *in vivo* effects of PFOA on tooth development remain unestablished. Future studies should include separate and combined exposure groups to determine whether the observed changes reflect individual, combined, or synergistic effects of 8:2 FTOH, its metabolite PFOA, 7:3 FTCA, and fluoride. Our bone fluoride measurements were included to document systemic fluorine burdens in mineralized tissues. Although bone and teeth are distinct, they can exhibit similar fluoride accumulation patterns under chronic fluoride exposure; in a dental fluorosis mouse model, fluoride levels in bone and teeth tracked closely. Bone and dental PFOA and 7:3 FTCA concentrations were not assessed in this proof-of-concept study, and mineralized tissues have largely been excluded from existing fluorotelomer toxicokinetic analyses.^31^ Future work should explicitly measure PFOA and 7:3FTCA, relevant metabolites, and fluoride in bone and teeth following 8:2 FTOH exposure to clarify their potential role in skeletal and dental effects.

Another key gap is that the recent perspective on FTOHs across the product life cycle summarizes environmental releases and potential exposure pathways^25^ — for example, emissions from textile production (implying worker inhalation risk) and generally low residential indoor-air risk — but does not report real-world human 8:2 FTOH external doses, internal measurements, or human toxicokinetics/bioavailability. Consequently, a quantitative external-to-internal comparison for 8:2 FTOH in humans is not currently possible, and our assessment of human relevance remains anchored to internal PFOA, which has been extensively characterized in occupational biomonitoring and thus provides the only practical cross-species internal-dose reference at present.

This study provides new insights indicating that 8:2 FTOH—and other PFAS capable of similar metabolic conversion—may represent an understudied source of fluorine accumulation *in vivo*. By identifying a novel pathway of fluoride bioaccumulation via 8:2 FTOH metabolism, this work underscores the need to reevaluate sources of environmental fluoride exposure and their broader implications for public health.

## Supplementary Material



## Data Availability

Data will be made available on request.

## Notes

Additional experimental details, including materials and methods and supporting data (DOC); The micro–Xray fluorescence spectroscopy, Stability assessment of 24-h aged 8:2 FTOH dosing solutions prepared in 0.5% CMC, Effects of systemic 8:2 FTOH administration on body weight changes in male and female mice, Evaluation region of microhardness testing, Effects of 8:2 FTOH dose and sex on PFOA, 7:3 FTCA, and PFOS levels in plasma, Effects of 8:2 FTOH dose and sex on fluoride levels in urine, plasma, and bone, Effect of 8:2 FTOH dose and sex on QLF levels, Elemental analysis of enamel affected by 8:2 FTOH using micro-XRF, Effect of 8:2 FTOH dose and sex on microhardness of enamel and dentin, Effects of 8:2 FTOH dose and sex on enamel and dentin volume, thickness, and mineral density, Amelogenin protein expression after 8:2 FTOH exposure, SEM images of dentin affected by 8:2 FTOH exposure, Elemental analysis of enamel and dentin affected by 8:2 FTOH using SEM-EDX, Micro-CT evaluation of mandibular incisors following fluoride or 8:2 FTOH exposure. (PDF)
