# Supplementary material for: Exposure to Per- and Polyfluoroalkyl Substances (PFAS) Causes Dental Developmental Anomalies: Underrecognized Risk of Fluoride Bioaccumulation
Source: Environ Sci Technol. 2026 Jun 22;60(26):18442–55. doi: 10.1021/acs.est.5c18166 (PMC13348020; doi:10.1021/acs.est.5c18166)
Supplement: Supplementary file 1 [file es5c18166_si_003.pdf]

Supplementary materials

Title: Exposure to Per- and Polyfluoroalkyl Substances (PFAS) Causes Developmental Dental Anomalies: Underrecognized Risk of Fluoride Bioaccumulation

Motoki Okamoto [a,b§], Shohei Yamashita [a§], Nanako Kuriki [a,b], Susanne Brueckner [a], Ria Achong-Bowe [a], Melanie Mendonca [a], Juliana Sanches Trevizol [a,f], Natsumi Fujiwara [c], Shin Nakamura [a], Satoru Shindo [a], Takumi Memida [a], Manabu Mizuhira [d], Navi Gill Dhillon [e], Xiaozhe Han [a], Toshihisa Kawai [a], Marília Afonso Rabelo Buzalaf [f], Eric T Everett [g] and Maiko Suzuki [a*]

Prepared for Environmental Science and Technology

Number of pages 15 (including this page):

Number of Figures: 11

Number of Table: 2

Materials and Methods for supplementary data

The micro–Xray fluorescence spectroscopy **(Micro-XRF)**

Elemental mapping analysis was done to reveal the constituent elements of whole maxillary incisors affected by systemic 8:2 FTOH exposure as well as their distribution. The maxillary incisors specimen was evaluated under dry conditions using an XRF spectrometer (M4 TORNADO, Bruker, Berlin, Germany) based on a previous report (Ref.). The basic parameters for the micro-XRF were as follows: voltage 50 kV; current 600 μA; pixel size 4 μm; exposure time 12 min. The elemental mapping and spectrogram was create using Esprit software (Bruker, Berlin, Germany), and the distribution of Fe according to intensity was displayed. The data obtained on each specimen's surface were shown as radiograph and elemental maps. This analysis was successful in visualizing enamel hypoplasia due to high-dose 8:2 FTOH exposure (Black arrows).

Okamoto M, *et al*. Microstructural Evaluation of the Mineralized Apical Barrier Induced by a Calcium Hydroxide Paste Containing Iodoform: A Case Report. *J Endod* **50**, 243-251 (2024).

Animals

This study was conducted in accordance with the ARRIVE guidelines 2.0, and the completed author checklist is provided as supplementary information. All animal procedures were performed in compliance with the institutional guidelines for the use of vertebrate animals. The animal protocol was approved by the Institutional Animal Care and Use Committees (IACUC) of Nova Southeastern University (Protocol No. 2023.02.MSuz1), which is accredited by the Association for Assessment and Accreditation of Laboratory Animal Care International (AAALAC). Proof of ethical approval is available upon request.

Specimen	Electrode potential (mV)	F (ng) calculated by regression curve based on mV recorded in the trap
0.5% CMC	200	below DL
0.5% CMC	203	below DL
8:2 FTOH in 0.5% CMC	199.9	below DL
8:2 FTOH in 0.5% CMC	202.6	below DL
8:2 FTOH in in 0.5% CMC spiked w. 1900 ng F-	22.3	1947.3
8:2 FTOH in in 0.5% CMC spiked w. 1900 ng F-	19.5	2178.1

Supplementary Table S1. Stability assessment of 24-hour aged 8:2 FTOH dosing solutions prepared in 0.5% CMC. Fluoride concentrations were measured in dosing solutions containing 8:2 FTOH (125 mg/kg body weight) prepared in 0.5% carboxymethylcellulose (CMC) after 24 hours of aging. Electrode potentials (mV) and corresponding calculated fluoride levels (ng), based on the regression curve for the mV recorded in the trap, are shown for unspiked and fluoride-spiked solutions. All unspiked samples were below the detection limit (DL < 0.02 ppm), confirming that 8:2 FTOH does not release measurable fluoride under these conditions, whereas fluoride-spiked controls demonstrated expected recoveries.

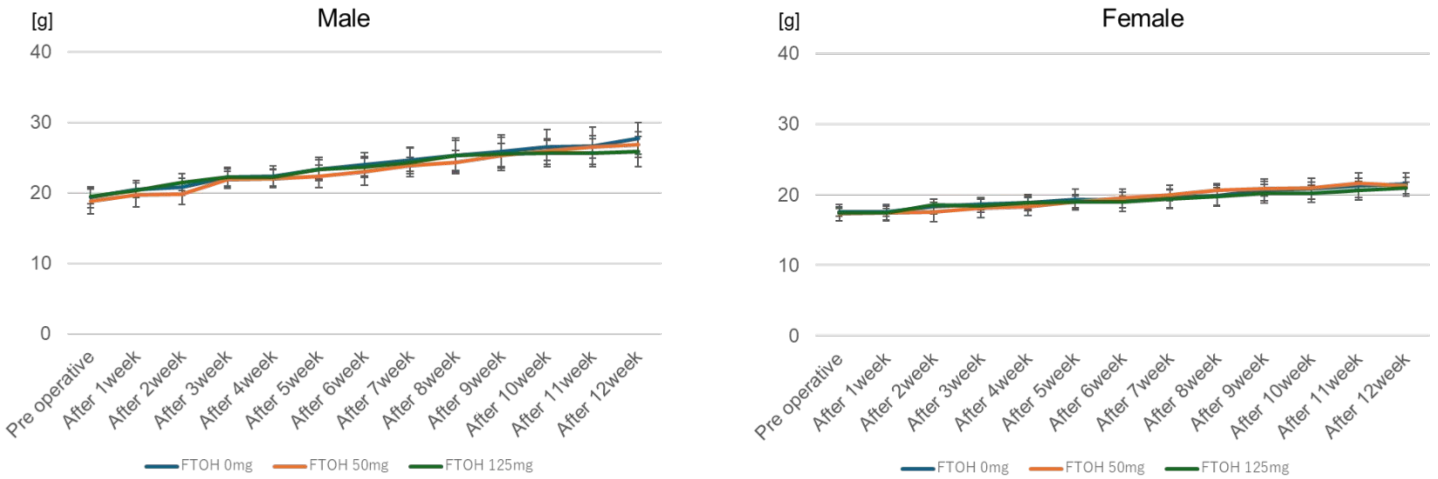

Supplementary Figure S1. Effects of systemic 8:2 FTOH administration on body weight changes in male and female mice

Both male and female mice continued to gain weight throughout the experiment. There were no abnormal changes in body weight during the administration period, and no significant differences were observed in the body weights among the groups. The results were analyzed using one-way ANOVA in each sex.

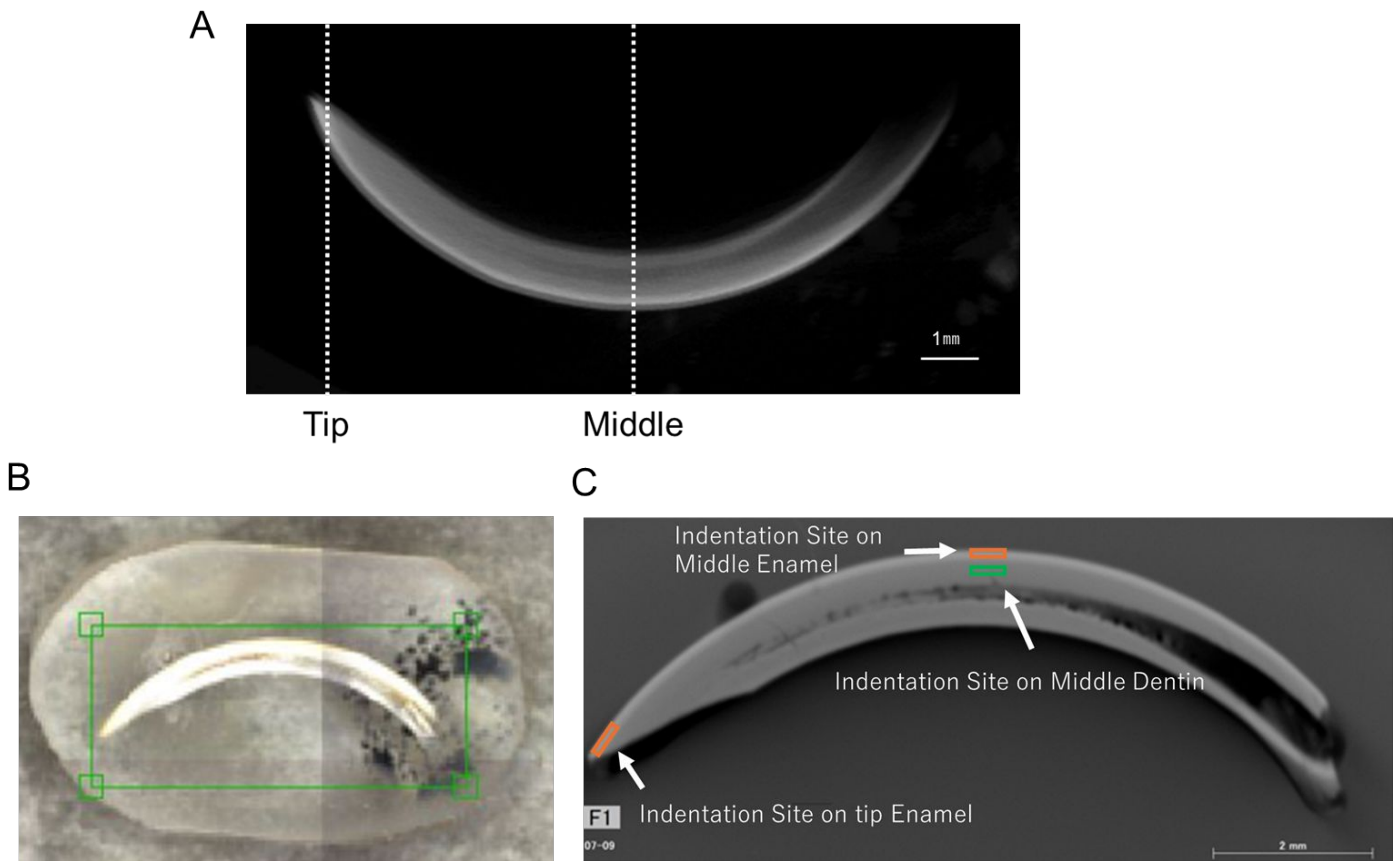

Supplementary Figure S2. Evaluation region of micro-hardness testing

A. Microhardness testing was performed on the tip and middle aspects. Indentation site in the middle region ranges 4-5 mm from the tip. The tip aspect was used to evaluate the enamel, and the middle aspect was used to evaluate the enamel and dentin. B. A representative image of a resin-embedded mouse mandibular incisor is shown. The mandibular incisor was polished to expose the observation surface. C. A radiograph of the same specimen is shown. Microhardness testing was performed at two sites on the enamel (apical and middle, orange box) and one site on the dentin (middle, green box).

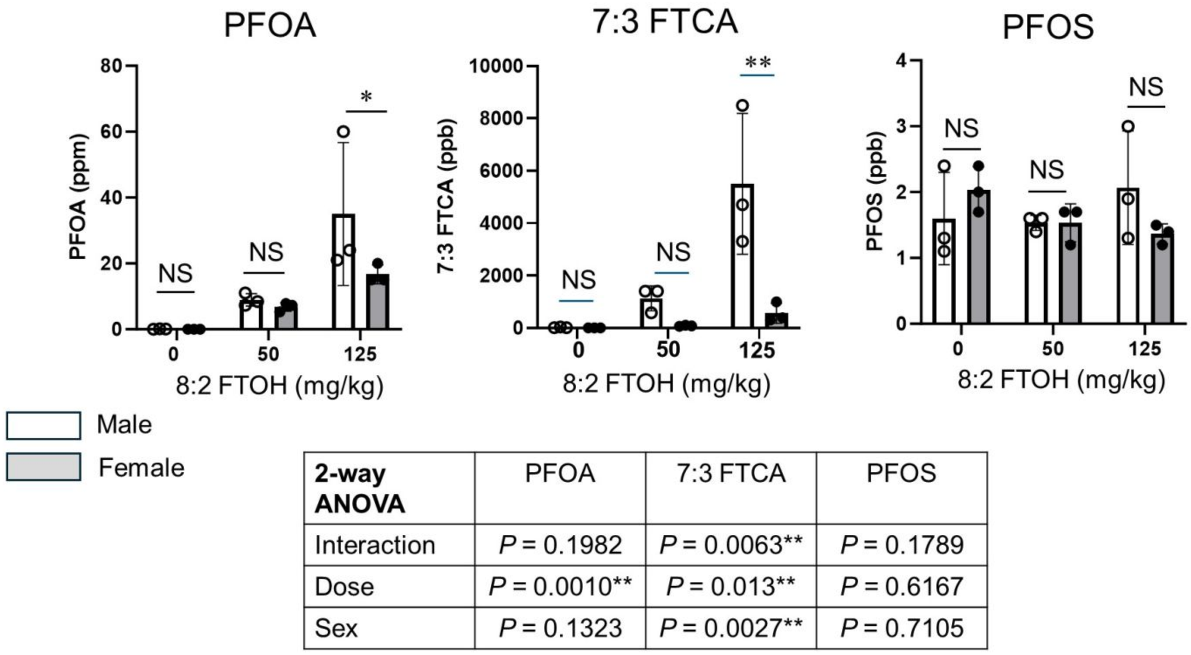

**2-way ANOVA**	PFOA	7:3 FTCA	PFOS
Interaction	*P* = 0.1982	*P* = 0.0063**	*P* = 0.1789
Dose	*P* = 0.0010**	*P* = 0.013**	*P* = 0.6167
Sex	*P* = 0.1323	*P* = 0.0027**	*P* = 0.7105

Supplementary Figure S3. Effects of 8:2 FTOH dose and sex on PFOA, 7:3 FTCA, and PFOS levels in plasma (Sub-analysis by two-way ANOVA).

Bar graphs show mean values (± SD) for PFOA (ppm, left panel), 7:3 FTCA (ppb, middle panel), and PFOS (ppb, right panel) across three doses of 8:2 FTOH (Ctrl, Low, and High dose). Males are represented by open circles (○), and females by filled circles (●). A significant increases in PFOA and 7:3 FTCA levels were observed at the 125 mg/kg dose (* $p < 0.05$), while PFOS levels did not significantly differ across doses. Two-way ANOVA revealed a significant main effect of

dose on PFOA levels (p = 0.0010), with no significant effects of sex or interaction. In contrast, 7:3 FTCA exhibited significant main effects of dose, sex, and their interaction. No significant effects were observed for PFOS.

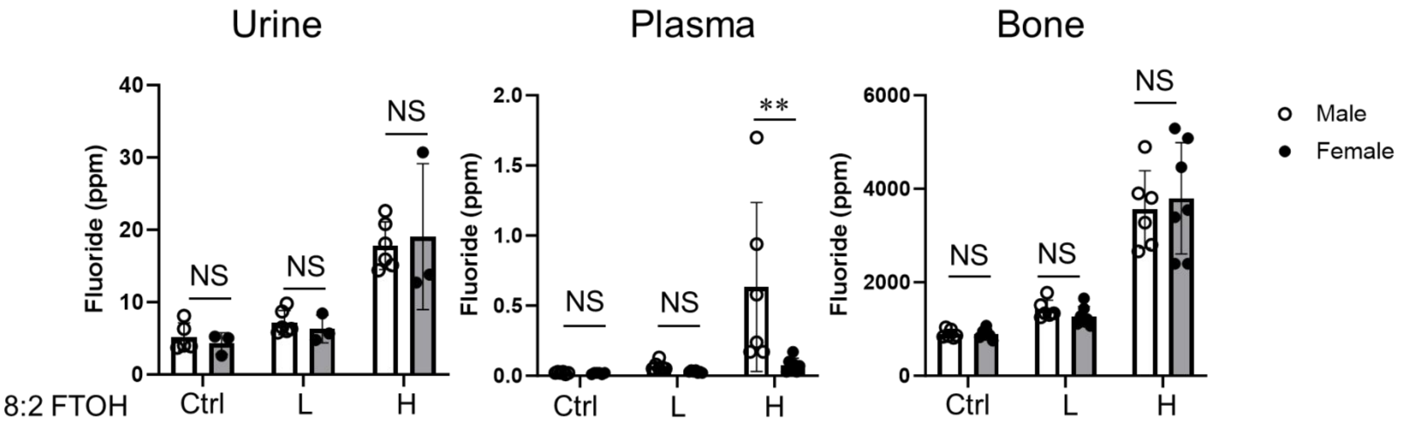

**2-way ANOVA**	Urine	Plasma	Bone
Interaction	P = 0.8185	P = 0.0105*	P = 0.7323
Dose	P < 0.0001**	P = 0.0019**	P < 0.0001**
Sex	P = 0.9129	P = 0.0150*	P = 0.8291

Supplementary Figure S4. Effects of 8:2 FTOH dose and sex on fluoride levels in urine, plasma, and bone (Sub-analysis by two-way ANOVA).

Bar graphs display fluoride concentrations (mean ± SD) in urine, plasma, and bone across three doses of 8:2 FTOH (Ctrl, low, and high dose). Males are represented by open circles (○), and females by filled circles (●). Two-way ANOVA revealed significant dose effects in all tissues (urine: $p < 0.0001$; plasma: $p = 0.0019$; bone: $p < 0.0001$). A significant interaction and sex effect were observed in plasma ($p = 0.0105$ and $p = 0.0150$, respectively), while no significant sex or interaction effects were found in urine or bone. Statistical significance is indicated by asterisks (* $p < 0.05$, ** $p < 0.01$), and "NS" denotes non-significant differences.

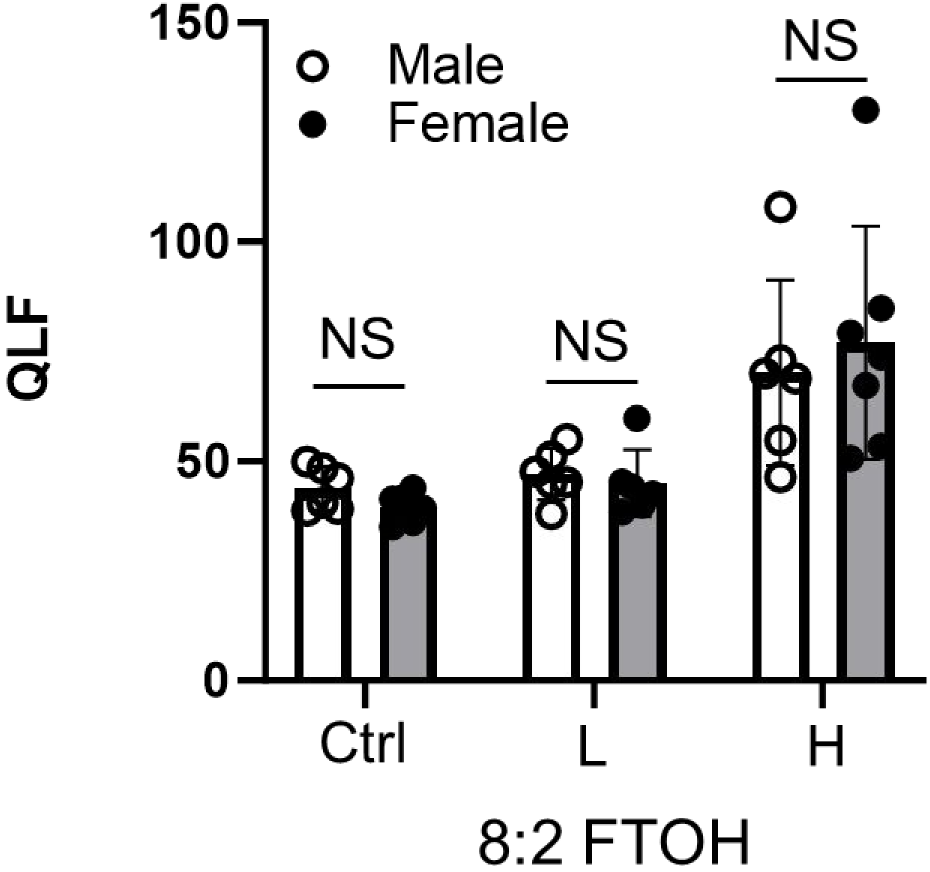

**2-way ANOVA**	QLF
Interaction	*P* = 0.6263
Dose	*P* = 0.0010**
Sex	*P* = 0.9700

Supplementary Figure S5. Effect of 8:2 FTOH dose and sex on QLF levels (Sub-analysis by two-way ANOVA). Bar graph displays QLF levels (mean ± SD) in male (open circles) and female (closed circles) subjects following administration of 8:2 FTOH at Control, Low-dose (L) and High-dose (H). A two-way ANOVA revealed a significant effect of dose ($p = 0.0010$), but no significant effect of sex ($p = 0.9700$) and no significant interaction between dose and sex ($p = 0.6263$). NS: no statistically significant difference between male and female groups at each dose level.

Ctrl

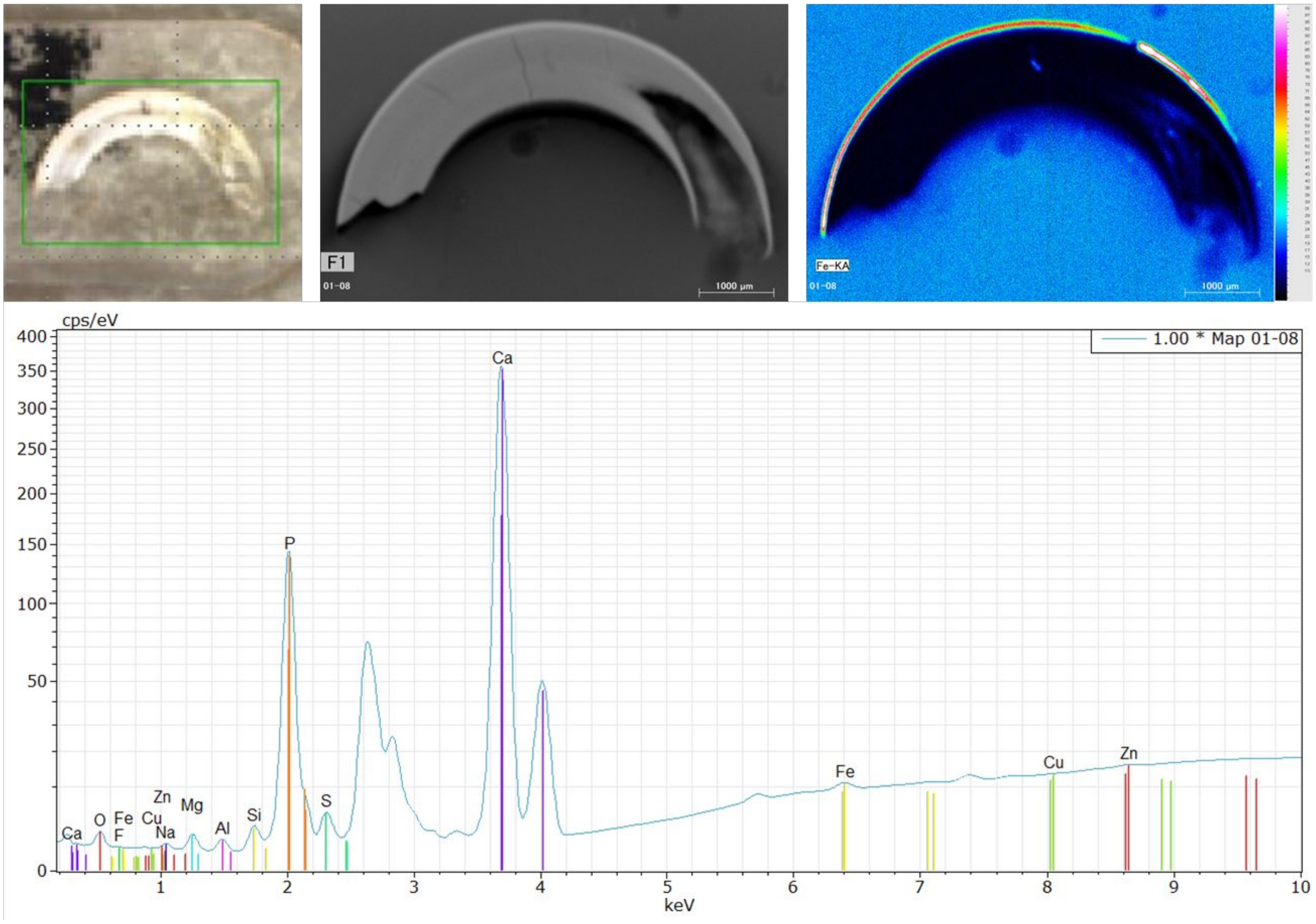

F1
01-08
1000 µm
Fe-KA
01-08
1000 µm
cps/eV
1.00 * Map 01-08
Ca
P
Ca
O
Fe
F
Cu
Zn
Na
Mg
Al
Si
S
Fe
Cu
Zn
keV

8:2 FTOH (L)

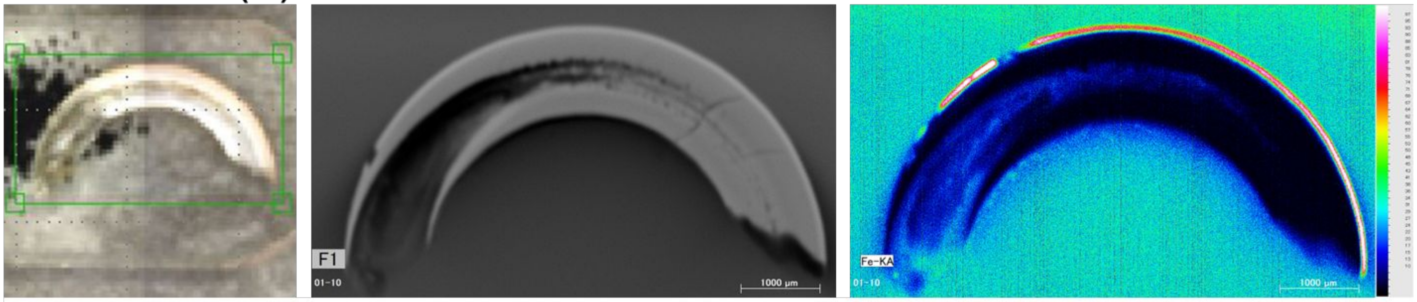
F1
01-10
1000 µm
Fe-KA
01-10
1000 µm

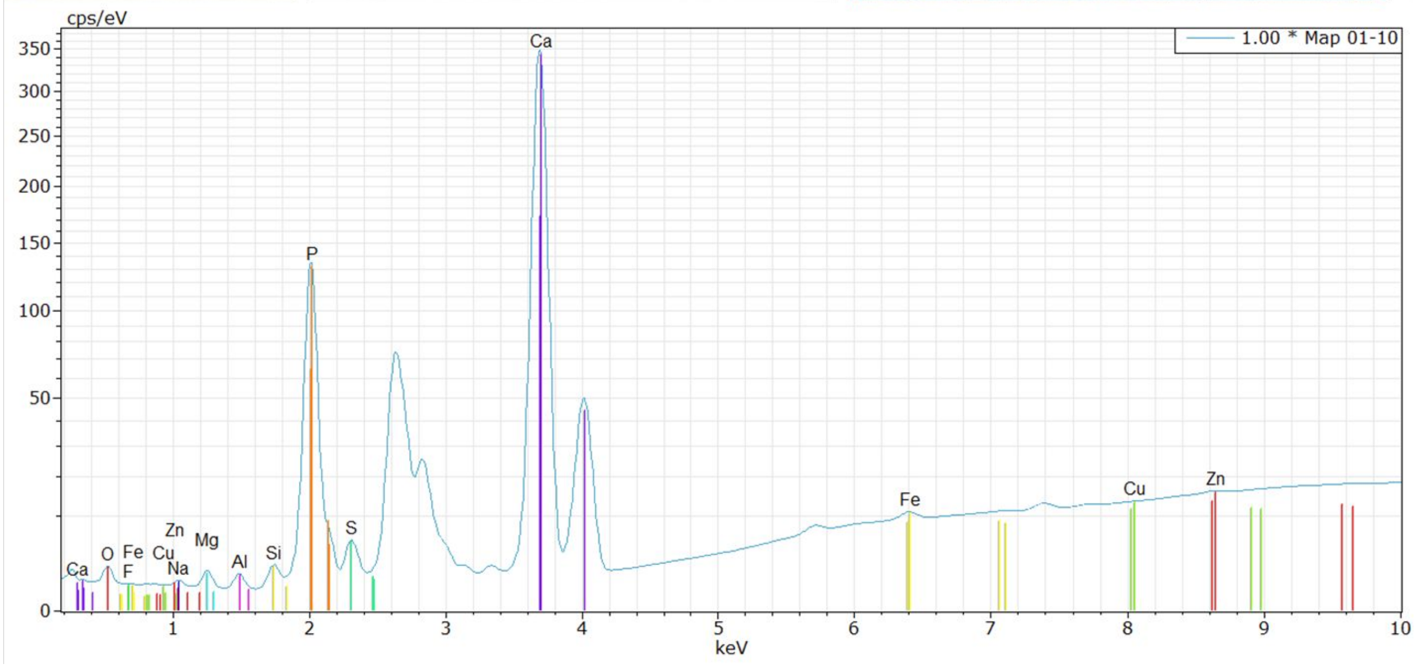
cps/eV
1.00 * Map 01-10
350
300
250
200
150
100
50
0
Ca
P
S
Ca
O
Fe
F
Cu
Zn
Na
Mg
Al
Si
Fe
Cu
Zn
1
2
3
4
5
6
7
8
9
10
keV

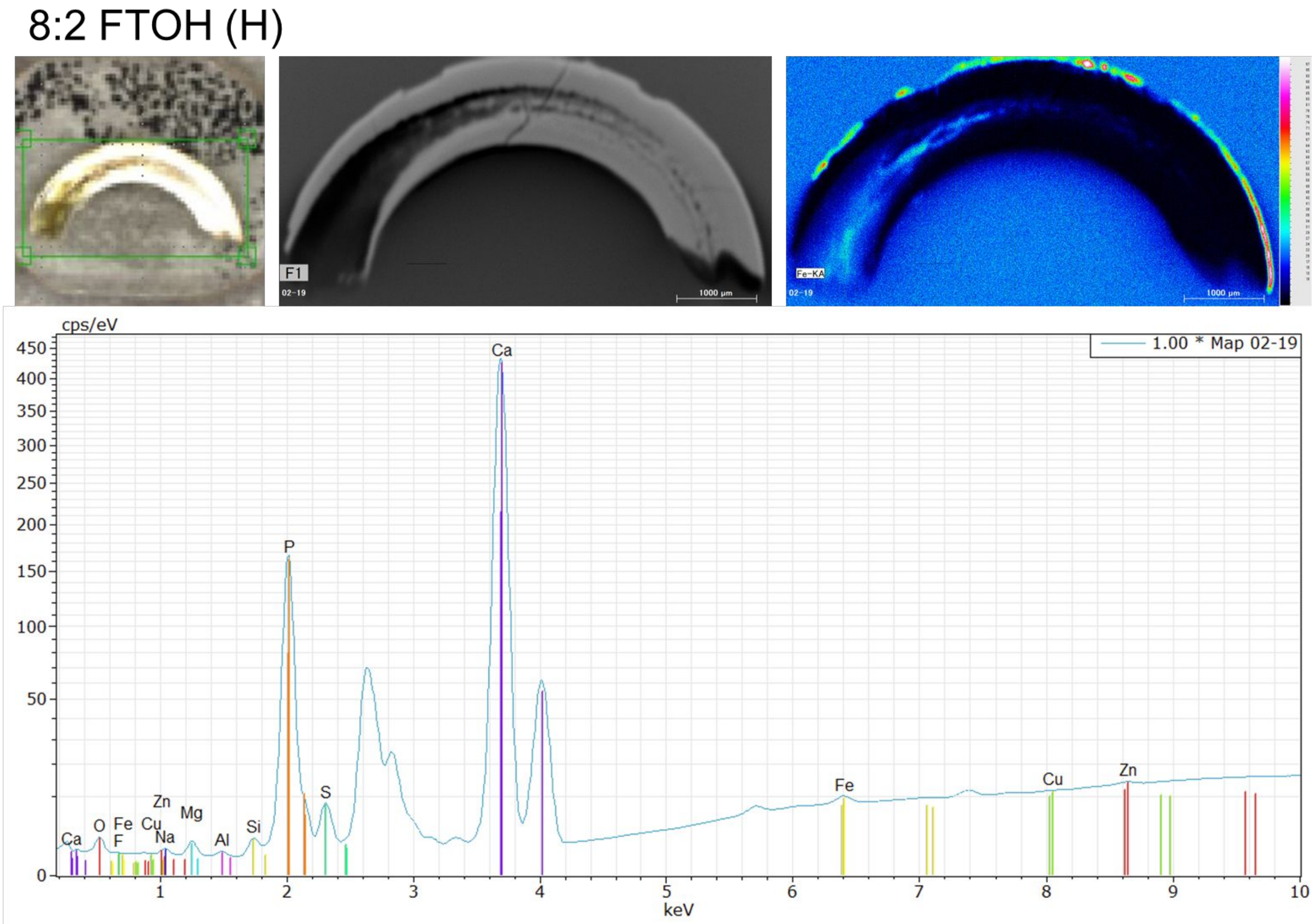

Supplementary Figure S6. Elemental analysis of enamel affected by 8:2 FTOH using micro-XRF.

The entire region of enamel in male maxillary incisors was analyzed using micro-XRF. Elemental mapping was conducted to evaluate the iron (Fe) distribution in enamel, which contributes to enamel pigmentation, mineralization and durability in mouse incisors. Heatmap of Fe reveals that 8:2 FTOH exposure disrupts Fe localization in enamel. Fe is involved in enamel maturation. XRF: X-ray

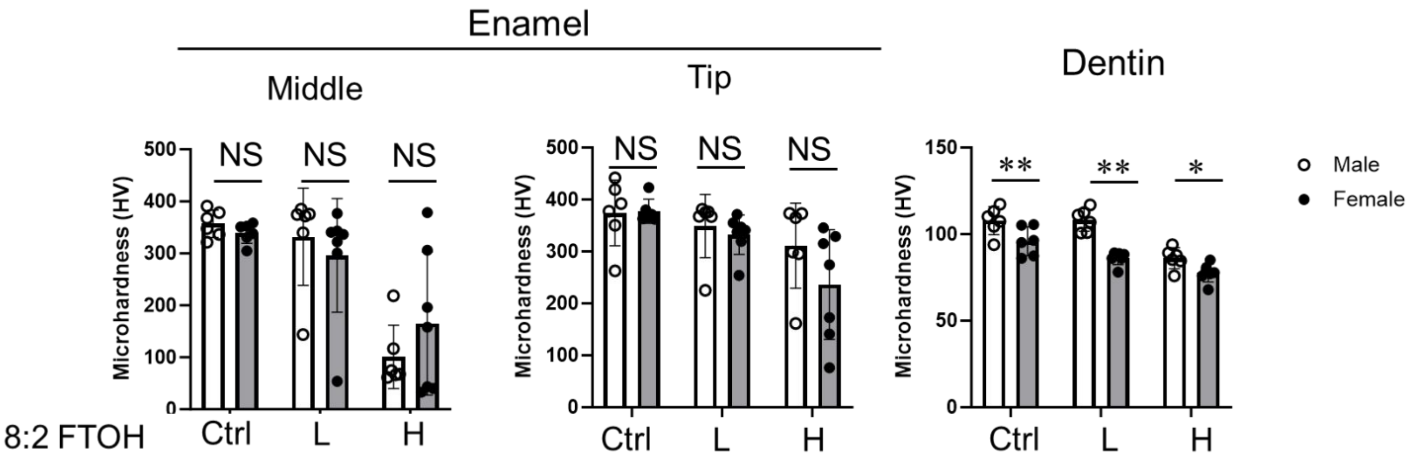

2-way ANOVA	Enamel		Dentin
	Middle	Tip	
Interaction	*P* = 0.3299	*P* = 0.3396	*P* = 0.0339*
Dose	*P* < 0.0001**	*P* = 0.0027**	*P* < 0.0001**
Sex	*P* = 0.9056	*P* = 0.1959	*P* < 0.0001**

Supplementary Figure S7. Effect of 8:2 FTOH dose and sex on microhardness of enamel and dentin (Sub-analysis by two-way ANOVA).

Microhardness of enamel and dentin in male and female subjects exposed to varying doses of 8:2 FTOH. Bar graphs show microhardness values (mean ± SD) measured at the middle and tip regions of the enamel, and in the dentin, for male (open circles) and female (closed circles) subjects. Two-way ANOVA results revealed significant effects of dose across all regions (middle enamel: **p** < 0.0001; tip enamel: **p** = 0.0027; dentin: **p** < 0.0001). A significant interaction between dose and sex was observed in dentin (**p** = 0.0339), but not in enamel regions. Sex had no significant effect on enamel microhardness, but was significant in dentin (**p** < 0.0001). Statistical significance is indicated by asterisks (* **p** < 0.05, ** **p** < 0.01), while "NS" denotes non-significant differences.

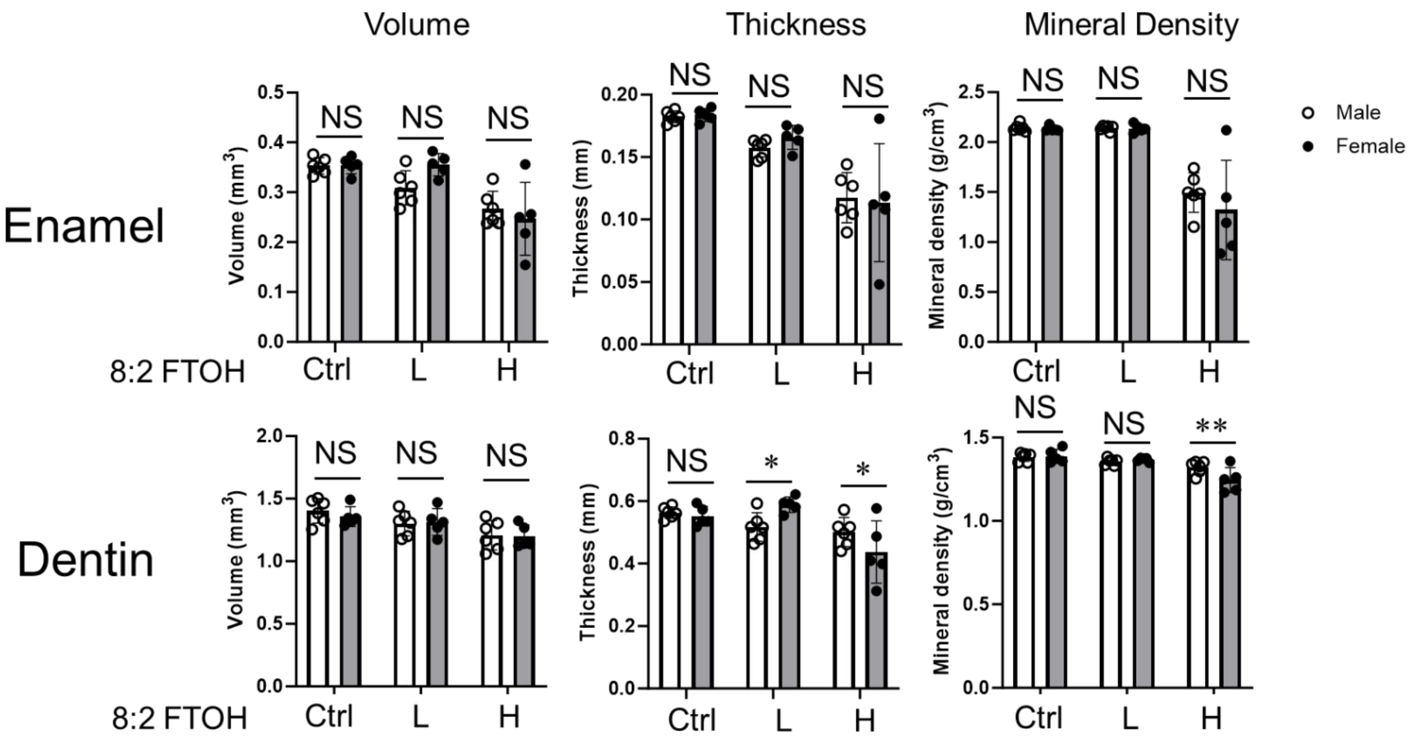

	2-way ANOVA	Volume	Thickness	Mineral Density
Enamel	Interaction	*P* = 0.1255	*P* = 0.7863	*P* = 0.5751
	Dose	*P* < 0.0001**	*P* < 0.0001 **	*P* < 0.0001**
	Sex	*P* =0.4909	*P* = 0.7899	*P* = 0.4031
Dentin	Interaction	*P* = 0.8012	*P* = 0.0145*	*P* = 0.0275*
	Dose	*P* = 0.0013**	*P* = 0.0004 **	*P* < 0.0001**
	Sex	*P* = 0.6796	*P* = 0.9402	*P* = 0.1571

Supplementary Figure S8. Effects of 8:2 FTOH dose and sex on enamel and dentin volume, thickness, and mineral density (Sub-analysis by two-way ANOVA).

Bar graphs show mean values (± SD) for enamel (top row) and dentin (bottom row) across three doses of 8:2 FTOH (Ctrl, Low-dose, and High-dose). Males are represented by open circles (○), and females by filled circles (●). Two-way ANOVA results are summarized in the table below the graphs. For enamel, dose had a significant effect on all parameters (**p** < 0.0001), with no significant effects of sex or interaction. For dentin, dose significantly affected all parameters (volume: **p** = 0.0013; thickness: **p** = 0.0004; mineral density: **p** < 0.0001), with significant interaction effects observed for thickness (**p** = 0.0145) and mineral density (**p** = 0.0275). Statistical significance is indicated by asterisks (* **p** < 0.05, ** **p** < 0.01), while "NS" denotes non-significant differences.

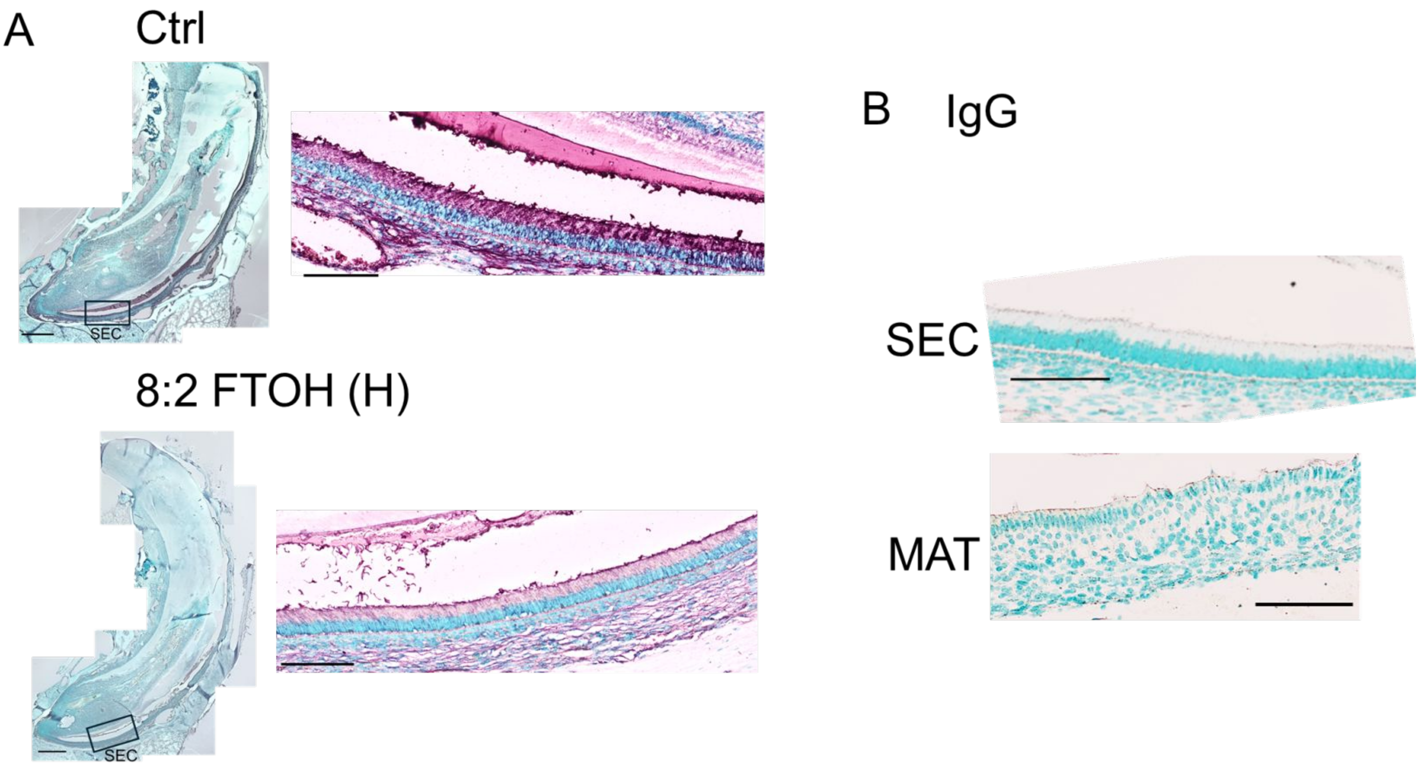

Supplementary Figure S9. Amelogenin protein expression after 8:2 FTOH exposure.

A. Representative images of Immunohistochemical staining (IHC) for amelogenin in male maxillary incisors after 8:2 FTOH exposure. Vehicle control (0 mg/kg; upper panel) and the high-dose (125 mg/kg; lower panel). Magnified images (right panels) of the boxed area of the secretory stage (SEC) are shown. Compared to control, a weak amelogening expression was observed in the ameloblast layer by 8:2 FTOH exposure. **B.** Representative images of IgG negative control in the secretory stage (SEC) and in the maturation stage (MAT). SEC: Secretory stage, MAT: Mature stage, Am: Ameloblast, SI: Stratum Intermedium, PL: Papillary layer.

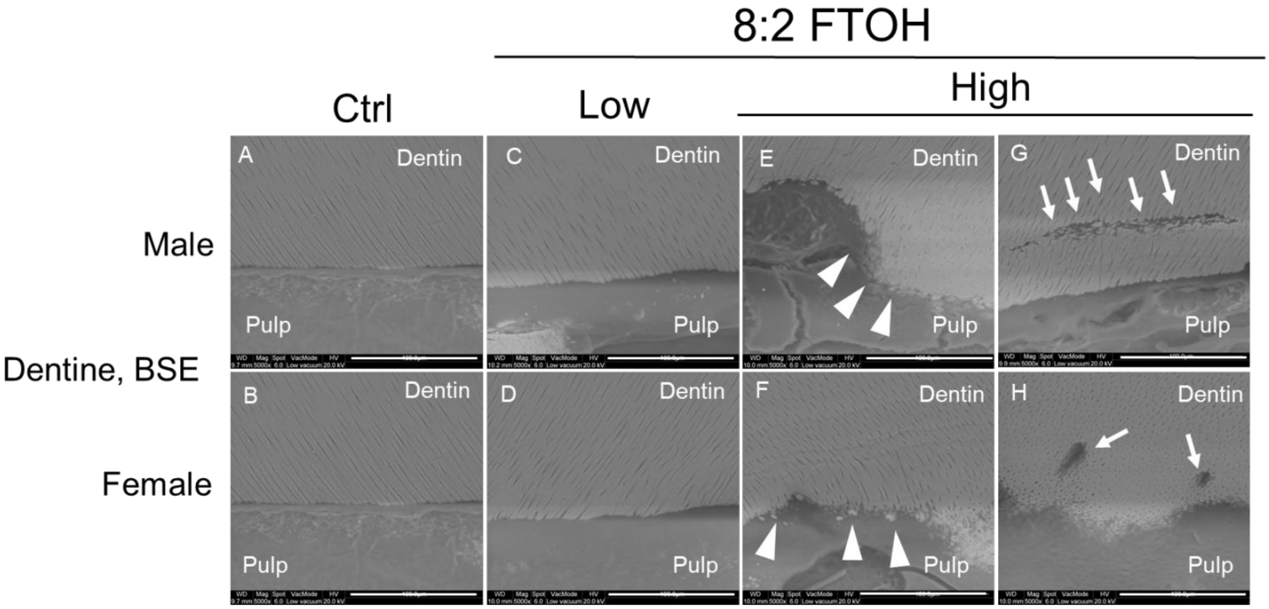

Supplementary Figure S10. SEM images of dentin affected by 8:2 FTOH exposure.

Representative BSE images of dentin from male and female mandibular incisors after 8:2 FTOH (Ctrl, low and high dose) exposures are shown. In high-dose 8:2 FTOH, dentin structure exhibited signs of impaired dentin hypoplasia, particularly at the boundary between the dentin and pulp tissue—the frontline region where odontoblasts are actively producing dentin. These alterations included impaired mineralization of matrix vesicles secreted by odontoblasts (arrowheads) and structural defects within the dentin (arrows). BSE: back scattering electron. Scale bars: 100 μM.

Elemental analysis of Enamel

[Wt%]

8:2FTOH	C	O	F	Na	Mg	P	Ca	Fe	Sr
Ctrl	39.9±3.6	31.4±0.9	0	0.33±0.02	0.14±0.03	9.0±1.2	19.2±2.3	0.04±0.03	0
Low	39.0±1.8	31.6±0.5	0	0.34±0.03	0.15±0.03	9.2±0.8	19.7±1.4	0.04±0.02	0
High	45.9±5.1*	30.0±2.0	0	0.38±0.05	0.12±0.02	7.9±1.1	16.8±2.4	0.06±0.02	0

Elemental Analysis of Dentin

[Wt%]

8:2FTOH	C	O	F	Na	Mg	P	Ca	Fe	Sr
Ctrl	34.6±1.4	35.9±1.9	0	0.51±0.05	0.59±0.15	9.6±0.7	18.8±1.5	0.01±0.01	0
Low	35.5±1.4	36.0±1.1	0	0.44±0.05	0.59±0.15	9.3±0.2	18.2±0.2	0.01±0.01	0
High	37.0±1.6*	34.8±0.7	0.02±0.03	0.49±0.05	0.38±0.06*	9.0±0.3	18.3±0.6	0.01± 0	0

Supplementary Table S2. Elemental analysis of enamel and dentin affected by 8:2 FTOH using SEM-EDX.

Middle region of male mandibular incisors was analyzed using SEM-EDX. Elemental mapping was conducted to show the distribution of elements in the enamel (upper panel) and dentin (lower panel). The analyzed elements included calcium (Ca), phosphorus (P), sodium (Na), magnesium (Mg), carbon (C), oxygen (O), and strontium (Sr). The Wt% of C in both enamel and dentin increased, and residual organic matter was thought to be the cause of enamel and dentin hypoplasia. In dentin, a decrease in Mg was also observed in the high dose- FTOH group compared to the control group.

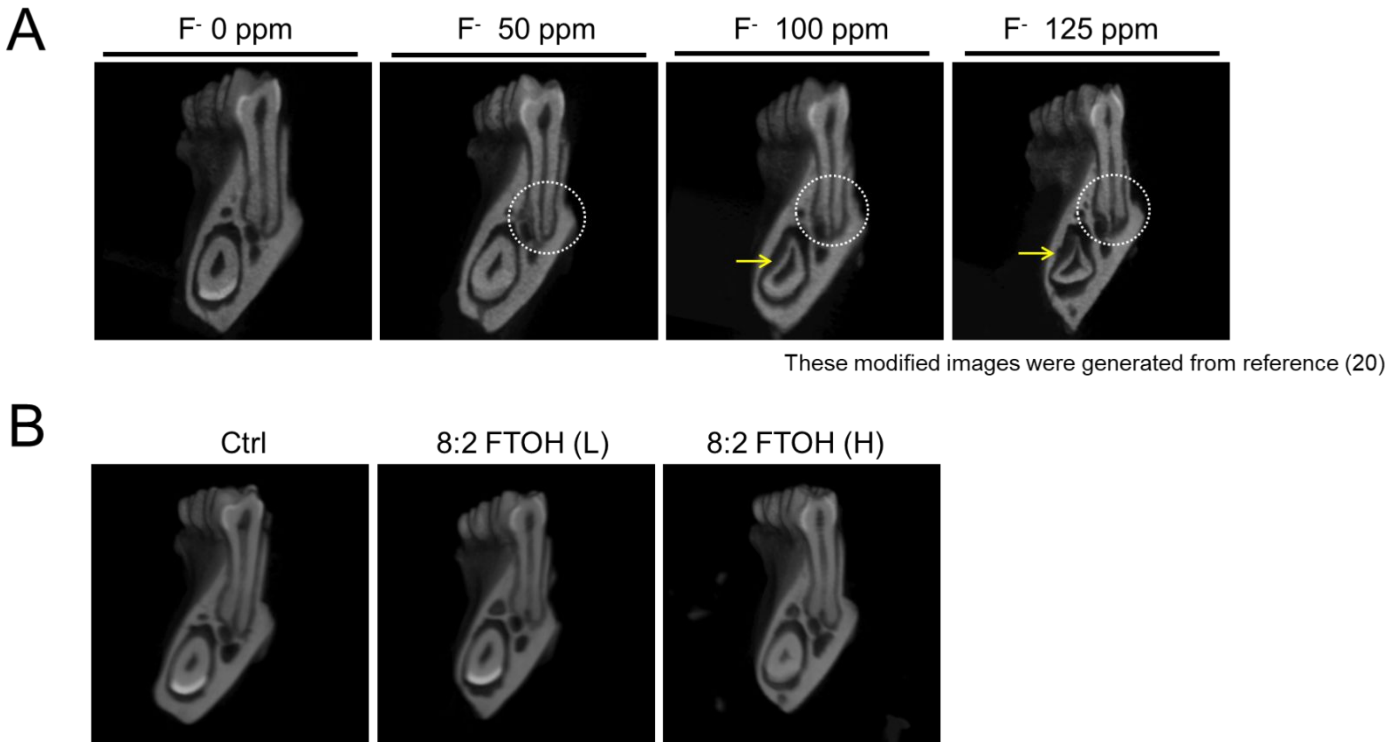

Supplementary Figure S11. Micro-CT evaluation of mandibular incisors following fluoride or 8:2 FTOH exposure. Representative 3D frontal cross-sectional images of mandibular incisors at the mesial roots of the mandibular first molars after treatment with fluoride (A, adapted from reference 20) or 8:2 FTOH (B). Arrows indicate the abnormal compressed, flattened triangular shape of the incisor. Circled areas highlight regions of root resorption in the mesial root of first molars.
